# Estimating global prevalence of gallbladder stones in general population from 2000 to 2024: systematic review and meta-analysis

**DOI:** 10.1080/07853890.2025.2570795

**Published:** 2025-10-10

**Authors:** Qingyang Ning, Fen Liu, Yiqiao Fang, Xixi Zhu, Jiaye Liu, Zhihui Li

**Affiliations:** ^a^ Division of Thyroid Surgery, Department of General Surgery, Division of Thyroid Surgery, Department of General Surgery, Laboratory of Thyroid and Parathyroid Diseases, Frontiers Science Center for Disease-Related Molecular Network, West China Hospital, Sichuan University, Chengdu, China; ^b^ Department of Respiratory and Critical Care Medicine, Frontiers Science Center for Disease-related Molecular Network, Center of Precision Medicine, Precision Medicine Key Laboratory of Sichuan Province, West China Hospital, Sichuan University, Chengdu, China; ^c^ Tianfu Jincheng Labratory, Frontiers Medical Center, Chengdu, China; ^d^ Department of Operating Room, West China Hospital, West China School of Nursing, Sichuan University, Chengdu, China

**Keywords:** Gallbladder stones, prevalence, general population, risk factor, health check

## Abstract

**Background:**

Gallbladder stones (GS), is one of the most common and costly of all the gastrointestinal diseases. However, global prevalence estimates of GS remain heterogeneous due to methodological variations across studies, and consensus on risk factor hierarchies is still evolving. Therefore, we performed current study in order to estimate the global prevalence of GS.

**Materials and methods:**

The quality of included studies was assessed using the Newcastle-Ottawa Scale. Data were analysed *via* the DerSimonian-Laird random-effects model with Logit transformations, and sensitivity analysis was performed using a ‘Leave-one-out’ approach.

**Results:**

Of 18,277 identified records, 139 studies were included in the final analysis. The overall global prevalence of GS in the general population was 5.86% (95% CI 5.28–6.47). Marked geographical disparities were observed, with the highest prevalence in Uganda (21.92%, 95% CI 18.43–25.61) and the lowest in Australia (0.18%, 95% CI 0.17–0.18) – a 122-fold difference. Multivariable meta-regression showed that study size was the strongest predictor (importance: 97.79%). Regarding risk factors, female gender, age > 50 years, increased body mass index, and family history of GS were significantly associated with higher GS prevalence. In contrast, factors such as education level, smoking, alcohol consumption, lifestyle, vegetarian diet, and serum lipid levels had no significant impact. Comorbidities including hypertension, diabetes mellitus, and metabolic-associated fatty liver disease (MAFLD) were strongly correlated with elevated GS prevalence.

**Conclusion:**

This meta-analysis showed that the GS was a common disease and affected the health of one in twenty people worldwide. Accurate estimates of the global and population-based prevalence of GS are helpful for healthcare improvements.

## Introduction

Gallbladder stones (GS) is a common disease of biliary system that often complicates with chronic or acute cholecystitis, cholangitis, pancreatitis, gallstone ileus, biliary tract obstruction, gallbladder empyema or perforation [1]. In western countries, approximately 5–25% adults suffer from GS, and 2–4% of them develop symptoms every year [2]. Patients with intractable symptoms or severe complications typically require cholecystectomy for radical treatment, imposing a substantial burden on healthcare resources – this translates to an annual expenditure of 5 billion US dollars globally [3,4]. More than 0.5 million cholecystectomies are performed in the US and 70,000 in England every year [5]. Accumulating evidence indicates that GS and its complications significantly elevate the risk of severe acute pancreatitis, diabetes, and cardiovascular disease, ultimately impairing patients’ quality of life and worsening prognosis [6–9].

Classic risk factors of GS include diabetes mellitus, persons who are obese, women, rapid weight cyclers, and patients on hormone therapy or taking oral contraceptives. Besides, ethnicity and nationality, which are closely correlated with eating habit, are often considered to be associated with GS prevalence [2,10]. The mechanism of GS formation is associated with supersaturation of biliary cholesterol due to hepatic hypersecretion, nucleation of cholesterol monohydrate crystals, and gallbladder hypomotility [11]. However, the full aetiology and pathogenesis of GS remain incompletely understood, likely influenced by a complex interplay of established risk factors and as-yet-unidentified contributors.

In this study, we aim to conduct a systematic review and meta-analysis to estimate the global prevalence of GS in the general population. Concurrently, we seek to synthesize evidence on risk factors associated with GS development from existing literature, with the goal of refining epidemiological understanding and informing targeted prevention strategies.

## Methods

### Data source and searching strategy

A comprehensive literature search was conducted across Embase, Medline, Web of Science, Cochrane Library, and Google Scholar databases to identify relevant studies published in English between January 2000 and February 2024. Eligible study designs included cohort studies, case series, and case-control studies investigating GS in the general population (defined as apparently healthy individuals). Reviews, editorials, letters, preprints, and conference proceedings were excluded. No trial registries were searched, and unpublished data were not sought. All searches were performed by a biomedical information specialist from the medical library, with a structured search strategy incorporating terms related to ‘gallbladder stones’, ‘prevalence’, and ‘general population’. Detailed search terms are provided in the supplementary material.

### Inclusion and exclusion criteria

Studies were included if they met the following criteria: (1) study design including cohort study, case series, or case-control study; (2) availability of complete data on GS prevalence; (3) study participants recruited from the general population; (4) study published in English. Studies were excluded if they failed to meet these criteria or fell into the following categories: (1) review articles, meta-analyses, abstracts, letters, or correspondence; (2) incomplete or unextractable GS prevalence data; (3) publication outside the January 2000–February 2024 timeframe.

### Data extraction

After removing duplicate records, two independent reviewers conducted initial screening based on titles and abstracts, adhering to predefined criteria. A random 10% of studies were cross-checked by an additional two investigators to ensure consistency. Full-text articles were then reviewed independently by two authors; discrepancies were resolved through consensus or, if necessary, consultation with a third team member. Extracted data included: publication year, World Health Organization (WHO) geographical region, country/regional income level (per World Bank classification), country/regional development status, study type, participant demographics (sex, age, body mass index [BMI]), lifestyle factors (smoking, alcohol consumption, education level), family history of GS, serum lipid profiles (total cholesterol [TC], triglycerides [TG], high-density lipoprotein cholesterol [HDL-C], low-density lipoprotein cholesterol [LDL-C]), and GS prevalence in participants with comorbidities. BMI was categorized as underweight (<18.5 kg/m^2^), normal weight (18.5–25 kg/m^2^), overweight (25–30 kg/m^2^), and obese (≥30 kg/m^2^) [12].

A study-specific data extraction form was used to standardize data collection, with one of four authors verifying extracted data against the original manuscripts for accuracy. Corresponding authors of studies with ambiguous, missing, or unspecified data were contacted *via* email for clarification; three studies were excluded due to unsuccessful contact with their corresponding authors. The Covidence platform was used to avoid data duplication. In cases where study results were published in multiple formats, data from the peer-reviewed article were prioritized.

### Quality assessment

Using the Newcastle-Ottawa Scale, which has three domains-selection, comparability and outcome, we evaluated the quality of included studies. The Newcastle-Ottawa Scale assigns a maximum score of five for selection, two for comparability, and two for outcome [13]. Studies were stratified into three quality tiers based on total NOS scores: low quality (1–3), moderate quality (4–6), and high quality (7–9). No studies were excluded based on quality scores to ensure transparency and comprehensive reporting of all relevant data.

### Statistical analysis

Meta‐analysis was carried out using the ‘Meta’, ‘Metafor’, and ‘Dmetar’ modules of the R‐4.2.2 statistical software suite. The main outcome for this study was the global prevalence of GS for general population. Individual sample proportion estimates and their 95% confidence intervals (CIs) were generated to determine the prevalence of GS in each country and region, and stabilized variances *via* the Logit transformation to approximate normal distribution. To quantify variance heterogeneity, random-effects models were fitted using the restricted maximum-likelihood estimation approach, and the Knapp-Hartung variance estimator was used to produce the 95% confidence intervals for summary measures. The percentage of variation that may be attributable to between-sample heterogeneity was quantified using the *I*^2^ statistic, with values higher than 75% indicating significant heterogeneity. A built-in function for ‘Leave-one-out’ analysis was used to do the sensitivity analysis. We re-estimated the pooling prevalence when outliers were found and the outlying studies were taken out of the calculation. We used univariable meta-regression of publication year, geographic region, income of countries or regions, country or region development, study size, and quality of research to evaluate temporal patterns in order to determine the impact of particular population-level variables on overall GS prevalence. In order to take into consideration important study-level characteristics affecting the overall GS prevalence, multivariable meta-regression utilizing the ‘dmetar’ package in R was used. Subgroup analysis was done to further explore the source of heterogeneity which estimated the pooled rate by dividing individuals into covariates. *p-*Value was used to compare the difference between subgroup analysis and *p* < 0.05 was considered as having significant difference.

## Results

### Study characteristic

**Figure 1.**
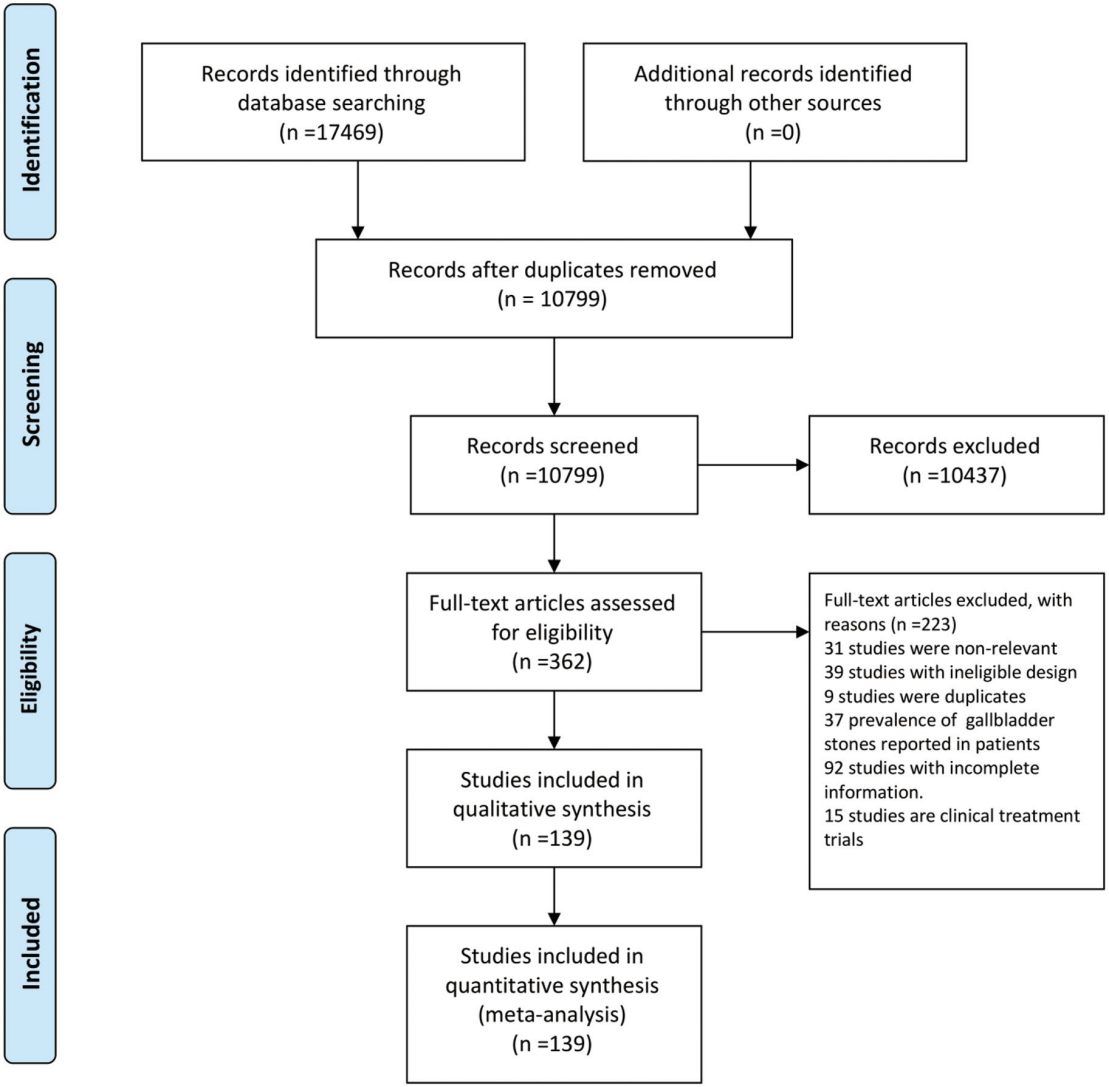
Study selection.

A total of 25,331 studies were retrieved from databases using the predefined search strategy. After removing duplicates, 18,277 studies were retained. Subsequent primary screening based on titles and abstracts led to the exclusion of 17,915 studies, and the remaining 362 studies underwent full-text screening for further eligibility assessment. Ultimately, 139 studies from 25 countries and regions (involving 21,868,822 participants) were included in the subsequent analysis (Figure 1). The geographic distribution of the included studies was as follows: Germany (*n* = 6), United States (*n* = 15), Argentina (*n* = 1), New Zealand (*n* = 1), Peru (*n* = 2), Bangladesh (*n* = 3), Iran (*n* = 6), Taiwan (China, *n* = 20), Japan (*n* = 8), Saudi Arabia (*n* = 2), Sweden (*n* = 2), South Korea (*n* = 15), Mainland China (*n* = 33), Italy (*n* = 2), Denmark (*n* = 5), Nigeria (*n* = 2), Mexico (*n* = 1), United Kingdom (*n* = 3), Uganda (*n* = 1), Ghana (*n* = 1), Ethiopia (*n* = 1), Australia (*n* = 1), Thailand (*n* = 1), and Russia (*n* = 1). Quality assessment was conducted for all included studies, revealing 114 high-quality studies and 25 fair-quality studies in this meta-analysis. Detailed characteristics of the included studies are presented in Supplementary Tables 1–2.

### Global prevalence of gallbladder stones in general population

**Figure 2.**
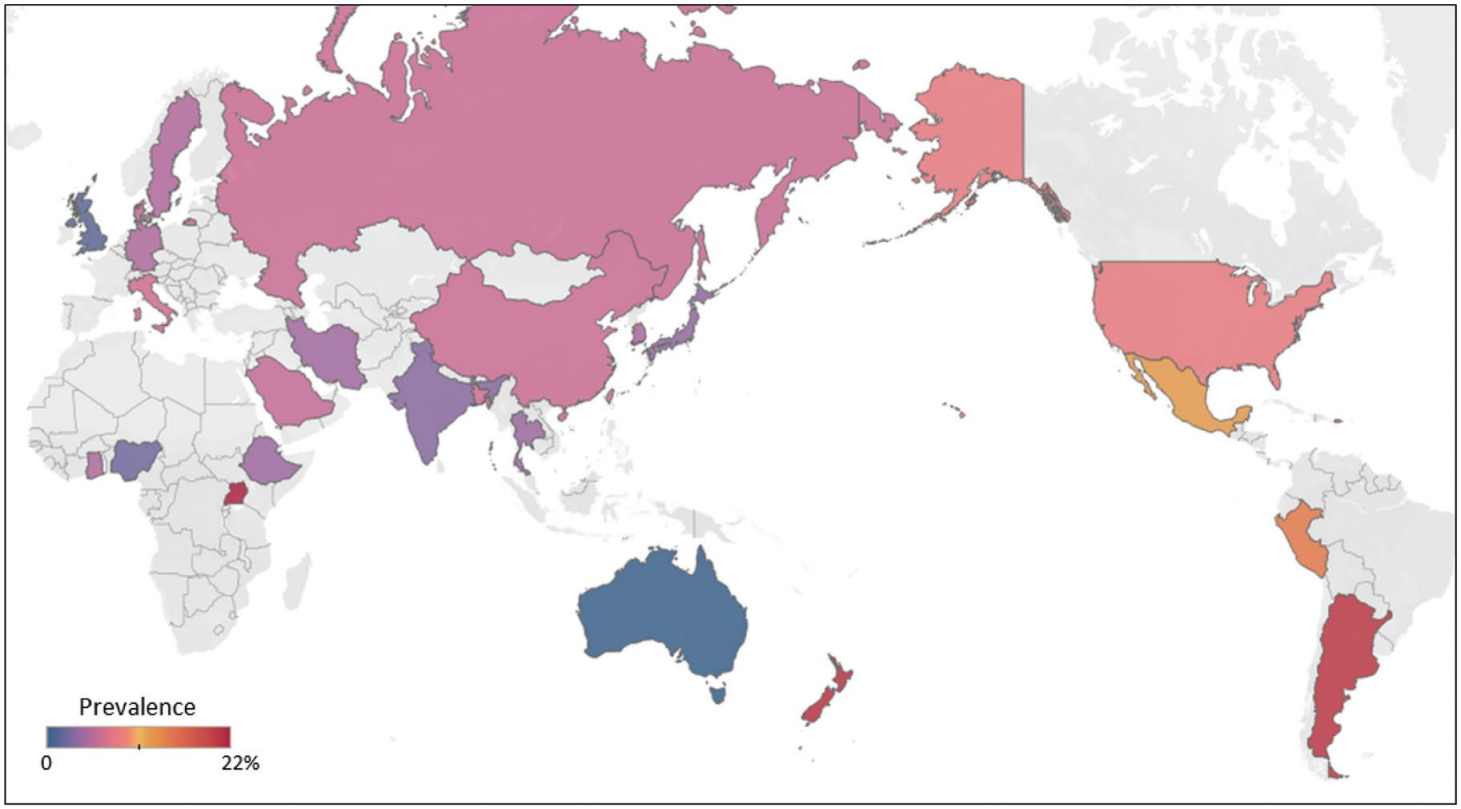
Global prevalence of gallbladder stones.

**Figure 3.**
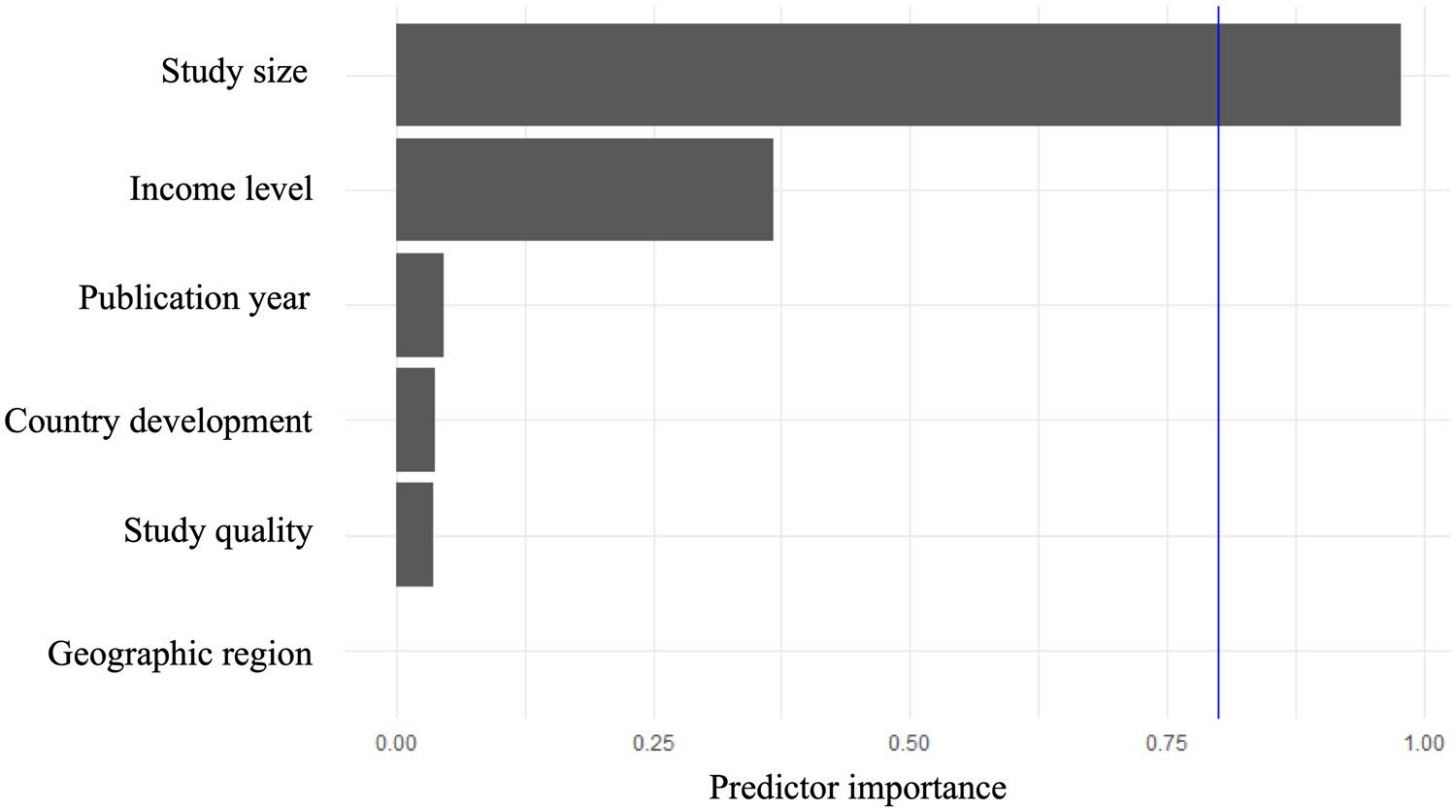
Multi-variable meta-regression for prevalence of gallbladder stones.

When we pooled data across all eligible studies, the overall prevalence of GS in general population was 5.86% (95% CI 5.28–6.47, *I*^2^ = 100.00%, Figure 2). A set of leave-one-out diagnostic tests were used for better understand the heterogeneity (Supplementary Table 3) and the results were further confirmed by using a build-in function in metafor (Supplementary Figure 1 and Table 4). Unfortunately, neither model was able to detect the outliers. Meta-regression analysis was carried out to further investigate the origin of heterogeneity. Our univariate meta-regression model indicated that publication year (*R*^2^ = 0.01, *p* = 0.20), quality score of study (*R*^2^ = 0, *p* = 0.79), development of countries or regions (*R*^2^ = 0.51, *p* = 0.44) were not significantly associated with heterogeneity. The source of heterogeneity across the studies, identified by meta-regression analyses, were study size (*R*^2^ = 0.01, *p* < 0.01), income of countries or regions (*R*^2^ = 0.50, *p* = 0.04) and geographical regions (*R*^2^ = 0, *p* < 0.01; Supplementary Table 5). By performing multivariable meta-regression, it was found that the study size with the highest predictor importance of 97.79% (Figure 3 and Supplementary Table 5)

### Subgroup analysis

To confirm the results from meta-regression, subgroup analysis was performed. When stratified by geographic regions, the prevalence of GS was 15.43% (95% CI 11.98–19.24, *I*^2^ = 93.80%), 8.44% (95% CI 5.06–12.60, *I*^2^ = 100.00%), 5.79% (95% CI 2.51–10.30, *I*^2^ = 97.80%), 5.57% (95% CI 4.85–7.33, *I*^2^ = 100.00%), 4.87% (95% CI 3.90–5.95, *I*^2^ = 99.80%), 4.78% (95% CI 1.94–8.78, *I*^2^ = 98.90%), 4.33% (95% CI 3.22–5.59, *I*^2^ = 89.40%) in Latin America and Caribbean, North America, Sub-Saharan Africa, East Asia and Pacific, Europe and Central Asia, Middle East and North Africa, South Asia, respectively (*p* < 0.01, Table 1). The GS prevalence differed significantly across countries (*p* < 0.01, Table 1), with Uganda having the highest prevalence (21.92%, 95% CI 18.43–25.61) and Australia having the lowest (0.18%, 95% CI 0.17–0.18). Based on income level of country or area categorized by World Bank evaluation, the pooled estimate prevalence of GS in high-, upper middle-, lower middle- and low income- country was 5.61% (95% CI 4.69–6.60, *I*^2^ = 100.00%), 6.99 (95% CI 6.22–7.80, *I*^2^ = 99.90%), 4.06% (95% CI 2.71–5.66, *I*^2^ = 97.80%), and 11.54% (95% CI 0.48–33.88, *I*^2^ = 99.20%), respectively (*p* < 0.01, Table 1). Unsurprisingly, the elderly (>50 years old) had an increasing GS prevalence (8.77%, 95% CI 7.39–10.26, *I*^2^ = 99.90%) than the younger (≤50 years old, 3.42%, 95% CI 2.45–4.54, *I*^2^ = 99.90%, *p* < 0.01, Table 1). Moreover, GS prevalence was 5.28% (95% CI 4.72–5.86, *I*^2^ = 99.90%) in male participants and significantly lower than that in female participants (7.33%, 95% CI 6.75–7.93, *I*^2^ = 99.90%, *p* < 0.01, Table 1). Studies with larger number of participants size (>10,000, 4.53%, 95% CI 3.78–5.33, *I*^2^ = 100.00%) had lower GS prevalence than studies with smaller ones (≤10,000, 7.05%, 95% CI 6.09–8.09, *I*^2^ = 100.00%, *p* < 0.01, Table 1). Nevertheless, study period did not show significantly association with GS prevalence (*p* = 0.07, Table 1). Furthermore, the prevalence of GS in high quality score studies (>7, 6.15%, 95% CI 4.45–8.11, *I*^2^ = 100.00%) was slightly higher than that in low or average score studies (≤7, 5.80%, 95% CI 5.08–6.56, *I*^2^ = 100.00%, but the difference was not significant (*p* = 0.73, Table 1).

**Table 1.** Subgroup analysis for prevalence of gallbladder stones in general population.

	Studies	GS	Participants	Prevalence (95% CI)	*p* Value	*I* ^2^
**Overall**	139	1040970	21868822	5.86 (5.28–6.47)	–	100.00%
**By region**					<0.01	
Latin America and Caribbean	4	1149	8173	15.43 (11.98–19.24)		93.80%
North America	15	45419	1159734	8.44 (5.06–12.60)		100.00%
Sub-Saharan Africa	5	369	6876	5.79 (2.51–10.30)		97.80%
East Asia and Pacific	79	936772	19498796	5.57 (4.85–7.33)		100.00%
Europe and Central Asia	19	32216	30247	4.87 (3.90–5.95)		99.80%
Middle East and North Africa	8	662	15898	4.78 (1.94–8.78)		98.90%
South Asia	8	630	14112	4.33 (3.22–5.59)		89.40%
**By country or area**					<0.01	
Uganda	1	112	511	21.92 (18.43–25.61)		–
New Zealand	1	66	318	20.75 (16.46–25.40)		–
Argentina	1	240	1173	20.46 (18.20–22.82)		–
Peru	2	300	2047	14.64 (13.14–16.21)		0.00%
Mexico	1	609	4953	12.30 (11.40–13.22)		–
United States	15	28643	928913	8.44 (5.06–12.60)		100.00%
Italy	2	2648	40368	6.86 (5.48–8.37)		96.40%
Bangladesh	3	226	3413	6.47 (4.94–8.19)		73.80%
Russia	1	107	1678	6.38 (5.26–7.60)		–
China Mainland	33	807901	15272831	6.34 (5.55–7.17)		100.00%
Saudi Arabia	2	43	696	6.07 (0.21–18.44)		96.20%
Denmark	5	18983	360020	6.03 (5.05–7.09)		99.40%
Taiwan	20	26632	793416	5.69 (4.21–7.37)		99.80%
Sweden	2	1708	58903	5.18 (1.10–11.94)		96.80%
Germany	6	607	8212	5.00 (2.56–8.19)		96.90%
Ghana	1	141	2824	4.99 (4.22–5.83)		–
South Korea	15	70070	1590703	4.89 (2.94–7.30)		100.00%
Iran	6	619	15202	4.41 (1.39–9.00)		99.20%
Ethiopia	1	68	1602	4.24 (3.31–5.29)		–
Thailand	1	141	3398	4.15 (3.50–4.85)		–
Japan	8	30080	774041	3.72 (3.05–4.46)		99.10%
India	5	404	10699	3.31 (2.50–4.23)		73.40%
Nigeria	2	48	1939	2.32 (1.30–3.61)		62.30%
United Kingdom	3	8117	360484	1.64 (0.81–2.75)		99.60%
Australia	1	1882	1064089	0.18 (0.17–0.18)		–
**By income level**					<0.01	
High income	81	230054	6546552	5.61 (4.69–6.60)		100.00%
Upper middle income	39	809298	15286080	6.99 (6.22–7.80)		99.90%
Lower middle income	17	1438	34077	4.06 (2.71–5.66)		97.80%
Low income	2	180	2113	11.54 (0.48–33.88)		99.20%
**By development**					0.29	
Developed	79	230011	6545856	5.60 (4.67–6.60)		100.00%
Developing	60	810959	15322966	6.21 (5.61–6.84)		99.90%
**By publication year**					0.07	
Before 2011	46	44076	759602	6.59 (5.66–7.57)		99.60
After 2011	93	996894	21109220	5.53 (4.84–6.26)		100.00%
**By study size**					<0.01	
≤10000	78	20986	261935	7.05 (6.09–8.09)		99.00%
>10000	61	1019984	21606887	4.53 (3.78–5.33)		100.00%
**By study quality score**					0.73	
≤7	25	287547	12445593	6.15 (4.45–8.11)		100.00%
>7	114	753423	9423229	5.80 (5.08–6.56)		100.00%
**By age**					<0.01	
≤50	30	297383	9026977	3.42 (2.45–4.54)		99.90%
>50	25	286192	3818246	8.77 (7.39–10.26)		99.90%
**By gender**					<0.01	
Male	103	460446	9745007	5.28 (4.72–5.86)		99.90%
Female	108	500346	9358197	7.33 (6.75–7.93)		99.90%
**By education**					0.51	
High school or below	15	34879	709006	6.51 (4.29–9.15)		99.90%
Collage or higher	15	14384	462890	5.56 (4.22–7.07)		99.60%
**By body mass index ^a^**					<0.01	
Underweight	10	15886	574277	2.42 (1.88–3.02)		96.00%
Normal weight	12	310837	6723260	4.96 (3.94–6.09)		99.90%
Overweight	12	184531	3246922	6.13 (3.57–9.30)		99.90%
Obesity	20	42440	743149	8.26 (5.50–11.49)		99.90%
**By smoke status**					0.34	
Smoker	40	35505	920209	5.95 (4.83–7.18)		99.80%
Nonsmoker	41	215564	3146123	7.75 (4.58–11.67)		100.00%
**By drink status**					0.09	
Drinker	31	49735	999683	5.57 (4.69–6.52)		99.70%
Nondrinker	31	106601	2025441	6.75 (5.90–7.65)		99.80%
**By lifestyle**					0.64	
Active	22	19723	349997	5.53 (4.05–7.21)		99.70%
Sedentary	22	39602	895724	6.01 (4.82–7.32)		99.80%
**By family history of GS**					<0.01	
Positive	4	100	667	16.55 (10.28–23.89)		72.90%
Negative	4	336	5528	7.81 (5.17–10.93)		91.30%
**By vegetarian**					0.90	
Vegetarian	3	327	16327	4.16 (0.79–9.62)		80.60%
Non-vegetarian	3	1189	38777	4.90 (2.24–8.52)		98.70%
**By TC ^b^**					0.44	
Normal	15	125412	1704276	5.56 (4.35–7.07)		99.90%
High	15	72434	776762	6.44 (4.83–8.53)		99.70%
**By TG ^c^**					0.27	
Normal	18	543483	10541974	5.46 (4.56–6.51)		99.90%
High	18	158033	2301910	6.43 (5.08–8.12)		99.90%
**By HDL-C ^d^**					0.16	
Normal	16	110482	1361892	5.60 (4.40–7.09)		99.90%
low	16	25028	258982	7.18 (5.59–9.19)		99.00%
**By LDL-C ^e^**					0.59	
Normal	11	93153	1143242	5.90 (4.63–7.48)		99.80%
High	11	37949	367953	6.54 (4.93–8.61)		99.60%

GS: gallbladder stone; TC: total cholesterol; TG: triglyceride; HDL-C: high dentsity lipoprotein cholesterol; LDL-C: low density lipoprotein cholesterol. ^a^BMI: underweight, <18.5; normal weight, 18.5-25; overweight, 25–30; obesity, >30; ^b^TC: normal, <5.18 mmol/L; high, ≥5.18 mmol/L. ^c^TG: normal, <1.70 mmol/L; high, ≥1.70 mmol/L. ^d^HDL-C: normal, ≥1.04 mmol/L; low, <1.04 mmol/L. e.LDL-C: normal, <3.37 mmol/L; high, ≥3.37.

### Risk factor analysis

We further performed a pooled analysis of relative risk factors in current study. In terms of body mass index (BMI), the pooled prevalence of GS increased linearly with BMI level. The GS prevalence in underweight, normal weight, overweight and obesity was 2.42% (95% CI 1.88–3.02, *I*^2^ = 96.00%), 4.96% (95% CI 3.94–6.09, *I*^2^ = 99.90%), 6.13% (95% CI 3.57–9.30, *I*^2^ = 99.90%) and 8.26% (95% CI 5.50–11.49, *I*^2^ = 99.90%), respectively (*p* < 0.01, Table 1). Intriguingly, our results revealed surprising results about lifestyle and hobbies on GS prevalence. Individuals with active lifestyle (5.53%, 95% CI 4.05–7.21, *I*^2^ = 99.70%) did not indicate a significant lower GS prevalence than population with sedentary lifestyle (6.01%, 95% CI 4.82–7.32, *I*^2^ = 99.80%, *p* = 0.64, Table 1). Insignificant difference was also observed in GS prevalence between smokers (5.95%, 95% CI 4.83–7.18, *I*^2^ = 99.80%) and non-smokers (7.75%, 95% CI 4.58–11.67, *I*^2^ = 100.00%, *p* = 0.34, Table 1). The GS prevalence was slightly lower among drinkers (5.57%, 95% CI 4.69–6.52, *I*^2^ = 99.70%) than non-drinkers (6.75%, 95% CI 5.90–7.65, *I*^2^ = 99.80%), but the difference was not significant (*p* = 0.09, Table 1). Moreover, we found that vegetarian (4.16%, 95% CI 0.79–9.62, *I*^2^ = 80.60%, *p* = 0.90) was insignificantly associated with lower GS prevalence (Table 1). Besides, participants with family history of GS had a high impact on GS prevalence (16.55%, 95% CI 10.28–23.89, *I*^2^ = 72.90%) than those without (7.81%, 95% CI 5.17–10.93, *I*^2^ = 91.30%, *p* < 0.01, Table 1). We collected the data on the association between TC, TG, HDL-C, LDL-C, which reflect serum lipid metabolism, and GS prevalence. Insignificant difference was obsereved between participants with normal TC (5.56%, 95% CI 4.35–7.07, *I*^2^ = 99.90%) and those with high TC (6.44%, 95% CI 4.83–8.53, *I*^2^ = 99.70%, *p* = 0.44, Table 1). Similar results also were reached between participants with normal TG (5.46%, 95% CI 4.56–6.51, *I*^2^ = 99.90%) and those with high TG (6.43%, 95% CI 5.08–8.12, *I*^2^ = 99.70%, *p* = 0.27), participants with normal HDL-C (5.60%, 95% CI 4.40–7.09, *I*^2^ = 99.90%) and those with low HDL-C (7.18%, 95% CI 5.59–9.19, *I*^2^ = 99.00%, *p* = 0.16), participants with normal LDL-C (5.90%, 95% CI 4.63–7.48, *I*^2^ = 99.80%) and those with high LDL-C (6.54%, 95% CI 4.93–8.61, *I*^2^ = 99.60%, *p* = 0.59) (Table 1).

Furthermore, we estimated pooled prevalence of GS in the participants with comorbidity in Table 2. Participants with hypertension (10.53%, 95% CI 8.41–12.85, *I*^2^ = 99.90%) had a significantly higher prevalence of GS than those without hypertension (6.14%, 95% CI 4.41–8.14, *I*^2^ = 100.00%, *p* < 0.01, Table 2). Relative to participants without diabetes mellitus (6.77%, 95% CI 5.37–8.32, *I*^2^ = 100.00%), participants who were diagnosed with diabetes mellitus (11.53%, 95% CI 9.38–11.85, *I*^2^ = 99.80%, *p* < 0.01) had twice the risk of developing GS (Table 2). People with metabolic-associated fatty liver disease (MAFLD; 7.66%, 95% CI 6.26–9.19, *I*^2^ = 98.70%) had only a slightly higher risk than those without MAFLD (5.06%, 95% CI 4.13–5.08, *I*^2^ = 99.30%, *p* < 0.01, Table 2). In contrast, there was no significant difference in GS prevalence between *Helicobacter pylori* infected (6.92%, 95% CI 4.91–9.24, *I*^2^ = 96.40%) and uninfected individuals (5.58%, 95% CI 3.37–8.30, *I*^2^ = 98.80%, *p* = 0.43, Table 2).

**Table 2.** Pooled prevalence of gallbladder stones in participants with comorbidities of general population.

Comorbidity	Studies	GS	Participants	Prevalence (95% CI)	*p* Value	*I* ^2^
Hypertension					<0.01	
Yes	28	52,197	827,146	10.53 (8.41–12.85)		99.90%
No	28			6.14 (4.41–8.14)		100.00%
Diabetes mellites					<0.01	
Yes	39	24,349	555,569	11.53 (9.38–13.85)		99.80%
No	39			6.77 (5.37–8.32)		100.00%
MAFLD					<0.01	
Yes	23	7657	113,136	7.66 (6.26–9.19)		98.70%
No	23			5.06 (4.13–6.08)		99.30%
*Helicobacter pylori* infection					0.43	
Yes	3	1028	15,706	6.92 (4.91–9.24)		96.40%
No	3			5.58 (3.37–8.30)		98.80%

MAFLD: metabolic dysfunction-associated fatty liver disease.

## Discussion

### Geographical and demographic factors

This study provides a comprehensive synthesis of evidence mapping the global prevalence of GS. Only a few review studies in specific regions, including China mainland [14], Europe [15] and the United States [16] reported a wide range of GS prevalence. Our results indicated that GS affected one out of twenty adults globally. This study has aided in pooling together data on the effects of geographic regions and income level of country or area related to GS prevalence worldwide as well as in deciphering the risk factor for GS. Furthermore, we found that the GS prevalence of some countries did not match that of their corresponding geographic regions. For example, the Latin America and Caribbean showed a far higher GS prevalence, but the highest GS rate was absent in Uganda, Sub-Saharan Africa. For unexpectedly high prevalence of GS, potential contributors may include genetic predispositions, distinct dietary patterns, and reduced physical activity levels among Ugandan populations. It should also be noted that the referenced GS prevalence data from Uganda were derived from a single hospital-based study, which might introduce over-representation of extreme values and thus partially account for this epidemiological deviation [17]. To be noted, the GS prevalence of low-income countries is at two folds higher than that of high, upper-middle and lower-middle countries. Moreover, older age and women are more likely to develop GS. Intriguingly, our results revealed surprising results about that education level, smoking, drinking, sedentary lifestyle and not strictly vegetarian are not associated with increased GS prevalence. For comorbidities, hypertension, diabetes mellitus and MAFLD represented high GS prevalence, while *Helicobacter pylori* infection was not among them.

We estimated the pooled prevalence of GS in all geographic regions specified by WHO closed virtually to overall prevalence, except the significantly deviated rate in Latin America and Caribbean (15.43%). The extremely deviated rates may be explained by ethnic and dietary habits, which were poor in fibre and rich in meat [18]. Meanwhile, we found that the country income level significantly affected the GS prevalence, but the country development did not exert an influence on it. Before 2000, it was generally believed that the prevalence of GS was significantly higher in Western countries (5.90–21.90%) than averaged about 4% in Asia [19]. But our results showed that the GS prevalence in Europe and Central Asia was close to that in South Asia, and East Asia and Pacific region, indicating that the disease spectrum of GS changed after 2000.

GS is rare in neonates and adolescent, but become increasingly more common in adulthood [20,21]. The present studies showed that, the older age was a significant risk factor of GS [10]. Due to long-term exposure to many extraneous risk factors, risk of gallstone formation may increase persistently. A previous study demonstrated that the incidence of gallstone disease increased from 0.21% per year at age < 40, until 1.39% per year at age > 60 [22]. Other study had found a 4- to 10-fold increase in GS risk after age 40 [23]. Meanwhile, symptoms of GS and severe complications increase with age, leading to cholecystectomy in more than 40% patients with age > 40 in Germany [24].

In our study, the overall prevalence of GS in female was slightly higher than that in male. A meta-analysis performed in Mainland China showed the prevalence of GS was 7.1% in male and 11.3% in female, which tendency was in conformity with our result [14]. The consequence reflects female are more likely to generate gallstones than male [18,25,26]. Current research indicated a variety of sex hormones affecting the formation of gallstones, what oestrogens can increase cholesterol secretion and diminish bile salt secretion, and progestins can reduce bile salt secretion and impair gallbladder leading to stasis [18,27]. Besides, the studies suggested that exogenous oestrogens, including the use of oral contraception and postmenopausal oestrogen therapy, was a significant risk factor for GS development [11,21,27]. Noteworthily, in Japan, risk of gallstone generation was increased by male sex [28,29].

Obesity is a well-established risk factor for GS, due to high-saturated fat depositions releasing cytokines through the inflammatory cascade that leads to gallbladder dysfunction [30,31]. BMI is the most common parameter for assessing the obesity [32]. In this study, prevalence of GS elevated linearly with increasing BMI, what was consistent with previously published findings that elevated BMI is associated with an increased risk of GS [33]. In obesity patients, amount of adipocytes release adiponectin which can increase insulin sensitivity and fatty acid oxidation, and discharge an anti-diabetic effect. The resulting hyperinsulinemia causes the liver to release excessive cholesterol and inhibits gallbladder movement, which promotes gallstone motility [32]. In addition, a further study found that the relationship between female gender and gallstone generous was stronger than in male gender, because male had more lean body mass than female [34].

Our study also estimated prevalence in population of GS with different lifestyle and hobbits. Interestingly, both smoking and drinking were unrelated to high GS prevalence. In several previous studies, smoking and drinking were risk factor with GS incidence [23,35–37]. Although this paradox might be caused by the fact that we did not stratified smoking and drinking doses, there were some studies that could support our results. A Japanese study demonstrated that adjusted ORs were 1.15 (95% CI 0.83–1.61) and 1.05 (95% CI 0.70–1.57) for current smokers consuming <25 and >25 cigarettes/day comparing with non-smokers [38]. There is no evidence and no physiological mechanism to support and explain the association between smoking and GS. In other study, ORs of alcohol drinking was 1.06 (95% CI 0.77–1.47), representing no relationship with GS [39]. Although many studies have suggested that physical activity could reduce the risk of GS [23,40], our global meta-analysis found that no significant association between exercise and GS incidence was demonstrated, that is in a line with a cross-section conducted by Sakuta [41]. Besides, fibre supplement had a protective effect in gallstone formation, but results showed that vegetarian was not associated with decreased GS prevalence. This insignificant difference might be due to the strict dietary regimen adopted by vegetarians [10]. Dyslipidemia was generally considered to be associated with GS formation, but our result demonstrated that dyslipidemia including high TC, high TG, low HDL-C, high LDL-C was not related to variational GS prevalence [42]. It is believed that free cholesterol in HDL is preferentially metabolized to bile acids rather than being secreted into bile as cholesterol, but there is no consensus about the relationship between TC, TG, LDL-C and risk of GS [43].

### Lifestyle and comorbidity factors

The comorbidities, including hypertension, diabetes mellitus, and MAFLD were demonstrated significant association with elevated GS prevalence. Previous studies have shown that hypertension is linked to GS through elevated leptin levels. Leptin can obstruct contraction of gallbladder wall and increase production of cholesterol supersaturated bile, leading to the gallstone formation [44,45]. Diabetes mellitus and insulin resistance were thought to affect cholesterol and bile salt metabolism, and cholesterol gallstone formation [46]. MAFLD is a new term for replacement of non-alcoholic fatty liver disease (NAFLD), which is strongly associated with GS, especially in population with age <50 [47,48]. Noticeably, the pathological mechanisms of these comorbidities are associated with insulin resistance, what was found to be a predictor of gallstone formation derived from a case-control study [49], thus it can be inferred that they are not associated with gallstones in isolation [41,45,50].

### Limitations

To our knowledge, this is a pioneering study to comprehensively estimate the global prevalence of GS in the general population, yet there are limitations to be addressed by further research: firstly, data on GS prevalence are absent in some countries and regions in this global meta-analysis (potentially affecting result accuracy), and high heterogeneity in some source data cannot be fully explained; secondly, while study size emerged as the strongest predictor, it may reflect uneven regional distribution of included studies – overrepresenting populations from developed nations and underrepresenting genetically distinct high-risk zones; thirdly, results may be impacted by the lack of uniform stratification criteria (e.g. daily smoking/alcohol consumption, exercise frequency and intensity); fourthly, although we sought to integrate multi-country data to assess additional factors on GS, most included studies were from general population cohorts, with some lacking comprehensive reporting of key variables (e.g. blood lipid profiles, blood pressure measurements), limiting our ability to conduct pooled analysis for these parameters; fifthly, while studies have found the FXR-OATP1A2 axis and ABCG8 genetic variants to be positively associated with GS risk [51–53], included participants were diagnosed with GS *via* health check-ups or clinical examinations (without access to genomic sequencing for genetic variant typing), so our meta-analysis excluded data on the association between genetic mutation types and GS prevalence; sixthly, gallstone type is critical for GS research, but defining gallstone components requires post-surgical detection, and all included studies were epidemiological (lacking gallstone type data); seventhly, to ensure data completeness and mitigate period-specific biases from lifestyle/dietary differences between historical and contemporary populations, we only included studies published in the last 25 years – this temporal constraint hinders comprehensive assessment of chronic risk factors (e.g. physical inactivity, suboptimal diet, obesity), as illustrated by a 2024 study showing persistently high GS prevalence in Chile (consistent with 1988 autopsy reports) amid rising obesity rates, suggesting obesity had limited influence while ABCG8 and TRAF3 germline variants may outweigh environmental factors in disease pathogenesis [54,55].

## Conclusion

The heterogeneous global prevalence of gallstones underscores the necessity for geographically tailored prevention strategies in high-risk regions. To lessen the burden of GS, policymakers should commit to raising awareness, making the best use of available resources, and coordinating the activities of multiple countries. Given the high prevalence of GS among general population, routine test should be considered especially in female, aged and individuals with a GS familiar history and those with elevated BMI. Knowledge of risk factor would potentially lead to improved monitoring for GS-related adverse events, decrease the incidence of severe adverse events.

## Supplementary Material

Supplementary File.docx

PRISMA_checklist_new.pdf

## Data Availability

Data will be made available on reasonable request from our corresponding author (Z. Li) for current study.
