# Supplementary material for: Estimating global prevalence of gallbladder stones in general population from 2000 to 2024: systematic review and meta-analysis
Source: Ann Med. 2025 Oct 10;57(1):2570795. doi: 10.1080/07853890.2025.2570795 (PMC12517418; doi:10.1080/07853890.2025.2570795)
Supplement: Supplementary File.docx [file IANN_A_2570795_SM5214.docx]

**Supplementary File to**

**Estimating global prevalence of gallbladder stones in general population from 2000 to 2024: systematic review and meta-analysis**

**Supplementary method. Searching strategy for global prevalence of gallbladder stones**

| **Database** | **via** | **References** | **After removing duplications** |
| --- | --- | --- | --- |
| Embase | Embase.com | 8798 | 7264 |
| Medline | Ovid | 3894 | 2712 |
| Web of science | Web of Knowledge | 3579 | 2834 |
| Cochrane Central Register of Controlled Trials | Wiley | 9060 | 5467 |
| **Total** |  | **25331** | **18277** |

**Embase-8798 refs**

('Gallstones'/de OR ('Gallstone' OR 'Gall Stones' OR 'Biliary Calculi' OR 'Gall Stone’ OR 'Common Bile Duct Calculi’ OR 'Common Bile Duct Gall Stone' OR 'Common Bile Duct Gallstones' OR 'Common Bile Duct Gallstone' OR 'Common Bile Duct Gall Stones'):ab,ti,kw) AND ('epidemiological data'/de OR 'epidemiology'/de OR 'geographic distribution'/de OR 'patient volume'/de OR prevalence/exp OR 'incidence'/de OR geography/de OR 'geographic names'/exp OR 'cross-sectional study'/de OR (epidemiolog* OR ((geograph* OR global*) NEAR/3 (distribut*)) OR (patient* NEAR/3 volume*) OR prevalen* OR incidenc* OR population-based* OR cross-sectional*):ab,ti,kw) NOT ((animal/exp OR animal*:de OR nonhuman/de) NOT ('human'/exp)) NOT ('case report'/de OR 'case report*':ti) NOT ([Conference Abstract]/lim) AND [english]/lim

**Medline-3894 refs**

(Gallstones/ OR ('Gallstone' OR 'Gall Stones’ OR’ Biliary Calculi' OR 'Gall Stone’ OR’ Common Bile Duct Calculi’ OR’ Common Bile Duct Gall Stone’ OR’ Common Bile Duct Gallstones’ OR’ Common Bile Duct Gallstone' OR 'Common Bile Duct Gall Stones'). ab,ti,kf) and (Epidemiological Monitoring/ OR epidemiology. fs. OR exp Incidence/OR exp Prevalence/ OR Incidence/ OR Geography/ OR exp Geographic Locations/ OR Epidemiologic Studies OR Cross-Sectional Studies/OR (epidemiolog* OR ((geograph* OR global*) ADJ3 (distribut*)) OR (patient* ADJ3 volume*) OR prevalence * OR incidence * OR population-based* OR cross-sectional*). ab,ti,kf.) NOT (exp Animals/ NOT Humans/) NOT (news OR congres* OR abstract* OR book* OR chapter* OR dissertation abstract*). pt. AND english.la.

**Web of science-3579 refs**

TS=((Gallstones OR Gallstone OR Gall Stones OR Biliary Calculi OR Gall Stone OR Common Bile Duct Calculi OR Common Bile Duct Gall Stone OR Common Bile Duct Gallstones OR Common Bile Duct Gallstone OR Common Bile Duct Gall Stones) AND (epidemiolog* OR ((geograph* OR global*) NEAR/2 (distribut*)) OR (patient* NEAR/2 volume*) OR prevalen* OR incidenc* OR population-based* OR cross-sectional*) NOT ((animal* OR rat OR rats OR mouse OR mice OR murine OR dog OR dogs OR canine OR cat OR cats OR feline OR rabbit OR cow OR cows OR bovine OR rodent* OR sheep OR ovine OR pig OR swine OR porcine OR veterinar* OR chick* OR zebrafish* OR baboon* OR nonhuman* OR primate* OR cattle* OR goose OR geese OR duck OR macaque* OR avian* OR bird* OR fish*) NOT (human* OR patient* OR women OR woman OR men OR man))) AND DT=(Article OR Review)

**Cochrane-9060 refs**

(((('Gallstones' OR ' Gallstones ‘OR 'Gall Stones' OR 'Biliary Calculi' OR 'Gall Stone' OR 'Common Bile Duct Calculi' OR 'Common Bile Duct Gall Stone' OR 'Common Bile Duct Gallstones' OR 'Common Bile Duct Gallstone' OR 'Common Bile Duct Gall Stones'))):ab,ti,kw) AND ((epidemiolog* OR ((geograph* OR global*) NEAR/3 (distribut*)) OR (patient* NEAR/3 volume*) OR prevalen* OR incidenc* OR (population NEXT/1 based*) OR (cross NEXT/1 sectional*)):ab,ti,kw)

**Supplementary Table 1. Characteristics for including studies.**

| **Author** | **Publication year** | **Country** | **Geographic region** | **Income level** | **Country development** | **Quality** | **Cases** | **Total** |
| --- | --- | --- | --- | --- | --- | --- | --- | --- |
| Kratzer W | 2021 | Germany | Europe and Central Asia | High | developed |  | 69 | 1909 |
| J A Simon | 2000 | United States | North America | High | developed |  | 682 | 9650 |
| A P Brasca | 2000 | Argentina | Latin America and Caribbean | Upper middle | developing |  | 240 | 1173 |
| Chapman BA | 2000 | New Zealand | East Asia and Pacific | High | developed |  | 66 | 318 |
| P L Moro | 2000 | Peru | Latin America and Caribbean | Upper middle | developing |  | 219 | 1513 |
| C E Ruhl | 2000 | United States | North America | High | developed |  | 757 | 5653 |
| S C Dhar | 2000 | Bangladesh | South Asia | Lower middle | developing |  | 57 | 1058 |
| S Massarrat | 2001 | Iran | Middle East and North Africa | Lower middle | developing |  | 74 | 1882 |
| V Singh | 2001 | India | South Asia | Lower middle | developing |  | 82 | 2649 |
| James E Everhart | 2002 | United States | North America | High | developed |  | 528 | 3296 |
| T K Ferguson | 2002 | United States | North America | High | developed |  | 24 | 572 |
| Shih-Wei Lai | 2002 | Taiwan, China | East Asia and Pacific | High | developed |  | 72 | 1028 |
| Masako Okamoto | 2002 | Japan | East Asia and Pacific | High | developed |  | 483 | 9946 |
| G A Hossain | 2003 | Bangladesh | South Asia | Lower middle | developing |  | 108 | 1336 |
| Kim-Choy Ng | 2003 | Taiwan, China | East Asia and Pacific | High | developed |  | 181 | 3053 |
| G Salinas | 2004 | Peru | Latin America and Caribbean | Upper middle | developing |  | 81 | 534 |
| Hidenari Sakuta | 2005 | Japan | East Asia and Pacific | High | developed |  | 39 | 965 |
| Henry Völzke | 2005 | Germany | Europe and Central Asia | High | developed |  | 358 | 3374 |
| Chien-Hua Chen | 2006 | Taiwan, China | East Asia and Pacific | High | developed |  | 168 | 3333 |
| Volker Kaechele | 2006 | Germany | Europe and Central Asia | High | developed |  | 10 | 493 |
| Chi-Ming Liu | 2006 | Taiwan, China | East Asia and Pacific | High | developed |  | 95 | 2386 |
| S A Abu-Eshy | 2007 | Saudi Arabia | Middle East and North Africa | High | developing |  | 34 | 291 |
| Toshiharu Fujita | 2007 | Japan | East Asia and Pacific | High | developed |  | 918 | 44129 |
| D Katsika | 2007 | Sweden | Europe and Central Asia | High | developed |  | 1666 | 58402 |
| Andrea M Kriska | 2007 | United States | North America | High | developed |  | 560 | 3143 |
| Yoosoo Chang | 2008 | South Korea | East Asia and Pacific | High | developed |  | 440 | 19503 |
| Hui Sun | 2009 | China | East Asia and Pacific | Upper middle | developing |  | 384 | 3573 |
| Thomas Walcher | 2009 | Germany | Europe and Central Asia | High | developed |  | 167 | 2129 |
| Davide Festi | 2010 | Italy | Europe and Central Asia | High | developed |  | 856 | 11229 |
| W Kratzer | 2010 | Germany | Europe and Central Asia | High | developed |  | 3 | 307 |
| Gangwar Richa | 2010 | India | South Asia | Lower middle | developing |  | 32 | 702 |
| Sang Soo Kim | 2011 | South Korea | East Asia and Pacific | High | developed |  | 173 | 4125 |
| Constance E Ruhl | 2011 | United States | North America | High | developed |  | 1010 | 14228 |
| Eric N Taylor | 2011 | United States | North America | High | developed |  | 14669 | 247255 |
| Sayeed Unisa | 2011 | India | South Asia | Lower middle | developing |  | 272 | 6548 |
| Corinna Koebnick | 2012 | United States | North America | High | developed |  | 766 | 510816 |
| Qing Xu | 2012 | China | East Asia and Pacific | Upper middle | developing |  | 2527 | 60064 |
| Hua-Li Yang | 2012 | China | East Asia and Pacific | Upper middle | developing |  | 419 | 11397 |
| Xin Huang | 2013 | China | East Asia and Pacific | Upper middle | developing |  | 267 | 49383 |
| Yu-Shan Mao | 2013 | China | East Asia and Pacific | Upper middle | developing |  | 543 | 10461 |
| Stefan Stender | 2013 | Danmark | Europe and Central Asia | High | developed |  | 4106 | 77679 |
| Sang-Wook Yi | 2013 | South Korea | East Asia and Pacific | High | developed |  | 6841 | 114562 |
| Yen-Chun Chen | 2014 | Taiwan, China | East Asia and Pacific | High | developed |  | 141 | 1721 |
| Bolanle O Ibitoye | 2014 | Nigeria | Sub-Saharan Africa | Lower middle | developing |  | 37 | 1283 |
| Jung-Hwan Kim | 2014 | South Korea | East Asia and Pacific | High | developed |  | 22 | 330 |
| I-Ching Lin | 2014 | Taiwan, China | East Asia and Pacific | High | developed |  | 734 | 11914 |
| Chao Shen | 2014 | China | East Asia and Pacific | Upper middle | developing |  | 541 | 5672 |
| Hsi-Che Shen | 2014 | Taiwan, China | East Asia and Pacific | High | developed |  | 860 | 6511 |
| Yu Takahashi | 2014 | Japan | East Asia and Pacific | High | developed |  | 694 | 15551 |
| Chung-Hung Tsai | 2014 | South Korea | East Asia and Pacific | High | developed |  | 447 | 8188 |
| Hong Yang | 2014 | China | East Asia and Pacific | Upper middle | developing |  | 542 | 23294 |
| Farhad Zamani | 2014 | Iran | Middle East and North Africa | Lower middle | developing |  | 51 | 6079 |
| Li Zhu | 2014 | China | East Asia and Pacific | Upper middle | developing |  | 1240 | 9455 |
| Alireza Ansari-Moghaddam | 2015 | Iran | Middle East and North Africa | Lower middle | developing |  | 40 | 1522 |
| Bo Kobberø Lauridsen | 2015 | Danmark | Europe and Central Asia | High | developed |  | 3886 | 67826 |
| Jun Lv | 2015 | China | East Asia and Pacific | Upper middle | developing |  | 28345 | 487373 |
| Amir Reza Radmard | 2015 | Iran | Middle East and North Africa | Lower middle | developing |  | 266 | 1494 |
| Madhusudan Saha | 2015 | Bangladesh | South Asia | Lower middle | developing |  | 61 | 1019 |
| Fen-Ming Zhang | 2015 | China | East Asia and Pacific | Upper middle | developing |  | 882 | 10016 |
| Kazuo Inui | 2016 | Japan | East Asia and Pacific | High | developed |  | 837 | 21550 |
| Yasir Mehmood | 2016 | Saudi Arabia | Middle East and North Africa | High | developing |  | 9 | 405 |
| Seungho Ryu | 2016 | South Korea | East Asia and Pacific | High | developed |  | 3341 | 83275 |
| Seungho Ryu | 2016 | South Korea | East Asia and Pacific | High | developed |  | 9417 | 396720 |
| Chang Hee Kwon | 2017 | South Korea | East Asia and Pacific | High | developed |  | 1426 | 46893 |
| Oh-Sung Kwon | 2018 | South Korea | East Asia and Pacific | High | developed |  | 821 | 20763 |
| Tong Liu | 2018 | China | East Asia and Pacific | Upper middle | developing |  | 4329 | 88947 |
| Faiza Qayyum | 2018 | Danmark | Europe and Central Asia | High | developed |  | 5957 | 100149 |
| Mei-Yan Xu | 2018 | China | East Asia and Pacific | Upper middle | developing |  | 995 | 17971 |
| Shengguang Yan | 2018 | China | East Asia and Pacific | Upper middle | developing |  | 429 | 5450 |
| Yan Zheng | 2018 | United States | North America | High | developed |  | 8518 | 129979 |
| Hsin-Yin Hsu | 2019 | Taiwan, China | East Asia and Pacific | High | developed |  | 1148 | 15671 |
| Bukunmi Michael Idowu | 2019 | Nigeria | Sub-Saharan Africa | Lower middle | developing |  | 11 | 656 |
| Sathiyamoorthy Jothy | 2019 | India | South Asia | Lower middle | developing |  | 16 | 600 |
| Hyung Sun Kim | 2019 | South Korea | East Asia and Pacific | High | developed |  | 27979 | 154463 |
| Fei-Lin Su | 2019 | Taiwan, China | East Asia and Pacific | High | developed |  | 1040 | 14555 |
| Pei-Yuan Su | 2019 | Taiwan, China | East Asia and Pacific | High | developed |  | 207 | 3190 |
| Heather A Ward | 2019 | Multicenter |  |  |  |  | 23753 | 334986 |
| Qiyun Gu | 2020 | China | East Asia and Pacific | Upper middle | developing |  | 94 | 2288 |
| Dan Huang | 2020 | South Korea | East Asia and Pacific | High | developed |  | 17167 | 704437 |
| Sen Tao Song | 2020 | China | East Asia and Pacific | Upper middle | developing |  | 274 | 4009 |
| Jiwen Wang | 2020 | China | East Asia and Pacific | Upper middle | developing |  | 168092 | 2068523 |
| Kazuya Higashizono | 2021 | Japan | East Asia and Pacific | High | developed |  | 23843 | 611930 |
| Zeinab Sadri | 2021 | Iran | Middle East and North Africa | Lower middle | developing |  | 173 | 3626 |
| Yang Song | 2021 | China | East Asia and Pacific | Upper middle | developing |  | 516994 | 10937993 |
| Hoyoung Wang | 2022 | South Korea | East Asia and Pacific | High | developed |  | 178 | 3038 |
| Jin Wang | 2022 | United States | North America | High | developed |  | 534 | 4739 |
| Xinhe Zhang | 2022 | China | East Asia and Pacific | Upper middle | developing |  | 6537 | 284129 |
| Yalan Zhang | 2022 | China | East Asia and Pacific | Upper middle | developing |  | 27463 | 318403 |
| Tien-Shin Chou | 2023 | Taiwan, China | East Asia and Pacific | High | developed |  | 199 | 2263 |
| Signe E J Hansen | 2023 | Danmark | Europe and Central Asia | High | developed |  | 4443 | 108438 |
| Li Jiang | 2023 | China | East Asia and Pacific | Upper middle | developing |  | 192 | 2378 |
| Aynur Unalp-Arida | 2023 | United States | North America | High | developed |  | 1277 | 9232 |
| Santiago Henao-Morán | 2014 | Mexico | Latin America and Caribbean | Upper middle | developing |  | 609 | 4953 |
| Chan Nie | 2023 | China | East Asia and Pacific | Upper middle | developing |  | 5328 | 72893 |
| Keng-Wei Liang | 2021 | Taiwan, China | East Asia and Pacific | High | developed |  | 938 | 21268 |
| Sailimai Man | 2022 | China | East Asia and Pacific | Upper middle | developing |  | 1269 | 58862 |
| Shridhar Dwivedi | 2021 | India | South Asia | Lower middle | developing |  | 2 | 200 |
| Wenxiang Li | 2021 | United Kingdom | Europe and Central Asia | High | developed |  | 6800 | 286731 |
| Hugh Shunsuke Colvin | 2022 | Japan | East Asia and Pacific | High | developed |  | 3092 | 62333 |
| Yuanjie Pang | 2021 | China | East Asia and Pacific | Upper middle | developing |  | 30783 | 510137 |
| Stella Nimanya | 2020 | Uganda | Sub-Saharan Africa | Low | developing |  | 112 | 511 |
| Adam Gyedu | 2015 | Ghana | Sub-Saharan Africa | Lower middle | developing |  | 141 | 2824 |
| AG Kebede | 2008 | Ethiopia | Sub-Saharan Africa | Low | developing |  | 68 | 1602 |
| [Oh-Sung Kwon](https://pubmed.ncbi.nlm.nih.gov/?sort=date&size=200&term=Kwon+OS&cauthor_id=32590752) | 2020 | South Korea | East Asia and Pacific | High | developed |  | 394 | 8908 |
| Xinyu Zhao | 2020 | China | East Asia and Pacific | Upper middle | developing |  | 2005 | 79809 |
| Guochong Jia | 2019 | China | East Asia and Pacific | Upper middle | developing |  | 1043 | 84366 |
| Young-Kyu Kim | 2019 | South Korea | East Asia and Pacific | High | developed |  | 355 | 7886 |
| Xu Li | 2019 | China | East Asia and Pacific | Upper middle | developing |  | 79 | 897 |
| Fei Wang | 2019 | China | East Asia and Pacific | Upper middle | developing |  | 2598 | 16299 |
| Jian-Han Chen | 2019 | Taiwan, China | East Asia and Pacific | High | developed |  | 94 | 8162 |
| Daniel Mønsted Shabanzadeh | 2018 | Danmark | Europe and Central Asia | High | developed |  | 591 | 5928 |
| Sabina Waniek | 2018 | Germany | Europe and Central Asia | High | developed |  | 46 | 582 |
| Yen-Ling Liu | 2018 | Taiwan, China | East Asia and Pacific | High | developed |  | 537 | 7066 |
| Qiao-Hua Qiao | 2017 | China | East Asia and Pacific | Upper middle | developing |  | 919 | 7853 |
| Ibinabo Ibiebele | 2017 | Austrilia | East Asia and Pacific | High | developed |  | 1882 | 1064089 |
| J-Y Wang | 2017 | Taiwan, China | East Asia and Pacific | High | developed |  | 407 | 6211 |
| T J McConnell | 2017 | United Kingdom | Europe and Central Asia | High | developed |  | 1182 | 49652 |
| Qian Zhu | 2016 | China | East Asia and Pacific | Upper middle | developing |  | 873 | 18291 |
| Min-Sun Kwak | 2015 | South Korea | East Asia and Pacific | High | developed |  | 1069 | 17612 |
| Jia Liu | 2014 | China | East Asia and Pacific | Upper middle | developing |  | 498 | 11200 |
| Yen-Chun Lee | 2014 | Taiwan, China | East Asia and Pacific | High | developed |  | 768 | 12033 |
| Jau-Yuan Chen | 2014 | Taiwan, China | East Asia and Pacific | High | developed |  | 23 | 1296 |
| Zhao-Yan Jiang | 2013 | China | East Asia and Pacific | Upper middle | developing |  | 203 | 1270 |
| Xian-Hua Liao | 2013 | China | East Asia and Pacific | Upper middle | developing |  | 296 | 2605 |
| Chia-Yen Dai | 2013 | Taiwan, China | East Asia and Pacific | High | developed |  | 115 | 1701 |
| Hsueh-Chou Lai | 2013 | Taiwan, China | East Asia and Pacific | High | developed |  | 6123 | 421039 |
| Li-Ying Chen | 2012 | China | East Asia and Pacific | Upper middle | developing |  | 918 | 7570 |
| Farrokh Seilanian Toosi | 2011 | Iran | Middle East and North Africa | Lower middle | developing |  | 15 | 599 |
| Kuan-Fu Liao | 2012 | Taiwan, China | East Asia and Pacific | High | developed |  | 12782 | 249015 |
| Paul J R Banim | 2010 | United Kingdom | Europe and Central Asia | High | developed |  | 135 | 24101 |
| I Halldestam | 2009 | Sweden | Europe and Central Asia | High | developed |  | 42 | 501 |
| Kaewalin Rungsinaporn | 2008 | Thailand | East Asia and Pacific | Upper middle | developing |  | 141 | 3398 |
| E Corazziari | 2008 | Italy | Europe and Central Asia | High | developed |  | 1792 | 29139 |
| Kristi L Storti | 2005 | United States | North America | High | developed |  | 1281 | 8010 |
| Chung-Jyi Tsai | 2005 | United States | North America | High | developed |  | 2356 | 45912 |
| Edmund J Bini | 2005 | United States | North America | High | developed |  | 962 | 13465 |
| S Kono | 2002 | Japan | East Asia and Pacific | High | developed |  | 174 | 7637 |
| Oleg V Reshetnikov | 2002 | Russia | Europe and Central Asia | Upper middle | developing |  | 107 | 1678 |
| E S Schernhammer | 2002 | United States | North America | High | developed |  | 11495 | 153784 |
| Kratzer W | 2021 | Germany | Europe and Central Asia | High | developed |  | 69 | 1909 |

**Supplementary Table 2. Characteristics of participants in including studies.**

| **Study** | **Gender** | | **Age** | | **Smoke status** | | **Drink stutas** | | **Body mass index** | | | | **Lifestyle** | |
| --- | --- | --- | --- | --- | --- | --- | --- | --- | --- | --- | --- | --- | --- | --- |
|  | **Male** | **Female** | **≤50** | **>50** | **Smoker** | **Nonsmoker** | **Drinker** | **Nondrinker** | **under weight** | **normal weight** | **over weight** | **obesity** | **Active** | **Sedentary** |
| Kratzer W | 21/941 | 41/968 | 28/1332 | 41/577 |  |  |  |  |  |  |  |  |  |  |
| J A Simon | 274/4787 | 408/4863 |  |  |  |  |  |  |  |  |  |  |  |  |
| A P Brasca | 72/465 | 168/708 |  |  |  |  |  |  |  |  |  |  |  |  |
| Chapman BA | 27/149 | 39/169 |  |  |  |  |  |  |  |  |  |  |  |  |
| P L Moro | 53/497 | 166/1016 |  |  |  |  | 67/555 | 152/958 |  |  |  |  |  |  |
| C E Ruhl | 208/2672 | 549/2981 |  |  |  |  |  |  |  |  |  |  |  |  |
| S C Dhar | 18/544 | 39/514 | 46/949 | 11/109 |  |  |  |  | 32/1006 |  |  | 25/52 |  |  |
| [S Massarrat](https://pubmed.ncbi.nlm.nih.gov/?sort=date&size=200&term=Massarrat+S&cauthor_id=11350555) | 13/1373 | 61/509 | 14/1102 | 75/780 |  |  |  |  |  |  |  |  |  |  |
| V Singh | 24/1271 | 58/1378 |  |  |  |  |  |  |  |  |  |  |  |  |
| James E Everhart | 218/1251 | 364/2045 |  |  |  |  |  |  |  |  |  |  |  |  |
| T K Ferguson |  | 24/572 |  |  |  |  |  |  |  |  |  |  |  |  |
| Shih-Wei Lai | 39/602 | 33/426 |  |  | 20/274 | 52/754 | 10/119 | 62/909 |  |  |  | 12/134 |  |  |
| Masako Okamoto | 255/4953 | 228/4993 | 182/4357 | 301/5590 |  |  | 231/5096 | 252/4850 |  |  |  |  |  |  |
| G A Hossain |  | 108/1336 |  |  |  |  |  |  |  |  |  |  |  |  |
| Kim-Choy Ng | 97/1711 | 84/1342 |  |  |  |  |  |  |  |  |  |  |  |  |
| G Salinas | 39/273 | 42/261 | 68/468 | 13/74 |  |  |  |  | 38/228 |  | 33/230 | 10/76 |  |  |
| Hidenari Sakuta |  |  |  |  | 25/449 | 14/516 | 28/822 | 11/143 |  |  |  |  | 30/738 | 9/227 |
| Henry Völzke | 160/1839 | 198/1535 |  |  |  |  |  |  |  |  |  |  |  |  |
| Chien-Hua Chen | 74/1592 | 94/1741 | 13/1233 |  |  |  |  |  | 1/114 | 69/1747 | 76/1271 |  |  |  |
| Volker Kaechele | 2/218 | 8/275 | 10/493 |  |  |  |  |  |  |  |  |  |  |  |
| Chi-Ming Liu | 42/1235 | 53/1151 | 30/1468 | 96/918 |  |  |  |  | 57/1400 |  | 38/623 | 31/363 |  |  |
| S A Abu-Eshy | 6/143 | 28/148 | 34/291 |  | 6/33 | 28/258 | 2/26 | 32/265 | 6/74 |  | 28/217 |  |  |  |
| Toshiharu Fujita |  |  | 602/36569 | 316/7560 |  |  |  |  |  |  |  |  |  |  |
| D Katsika | 1076/30710 | 590/27692 |  |  | 169/7277 | 1330/43419 | 271/12674 | 1153/34732 |  | 878/33425 | 450/10600 | 70/1623 |  |  |
| Andrea M Kriska | 212/1187 | 348/1956 |  |  | 383/2215 |  |  |  |  |  |  |  |  |  |
| Yoosoo Chang |  |  |  |  | 310/13820 | 130/5683 |  |  |  |  |  |  | 279/11705 | 161/7798 |
| Hui Sun | 181/1825 | 203/1748 | 89/1622 |  |  |  |  |  | 281/2841 |  | 103/732 |  |  |  |
| Thomas Walcher | 49/1025 | 118/1104 | 56/1455 | 101/674 | 81/1154 | 85/973 | 95/1493 | 67/589 |  |  |  |  |  |  |
| Davide Festi |  |  |  |  |  |  |  |  |  |  |  |  |  |  |
| W Kratzer | 2/150 | 1/157 |  |  |  |  |  |  |  |  |  |  |  |  |
| Gangwar Richa |  | 32/702 |  |  |  |  |  |  |  |  |  |  |  |  |
| Sang Soo Kim |  | 173/4125 |  |  | 7/260 | 166/3865 |  |  |  |  |  |  |  |  |
| Constance E Ruhl |  |  |  |  |  |  |  |  |  |  |  |  |  |  |
| Eric N Taylor | 2004/51473 | 12665/195782 |  |  |  |  |  |  |  |  |  |  |  |  |
| Sayeed Unisa | 52/2625 | 220/3923 |  |  |  |  |  |  |  |  |  |  |  |  |
| Corinna Koebnick | 148/252349 | 618/258467 | 766/510816 |  |  |  |  |  | 215/301549 |  | 179/99987 | 372/109280 |  |  |
| Qing Xu | 1290/32380 | 1237/27684 | 1012/37176 | 1515/18920 |  |  |  |  | 1120/32540 |  | 1407/23556 |  |  |  |
| Hua-Li Yang |  |  |  |  |  |  |  |  |  |  |  |  |  |  |
| Xin Huang |  |  |  |  |  |  |  |  |  |  |  |  |  |  |
| Yu-Shan Mao | 343/7331 | 200/3130 | 195/6420 | 348/4041 |  |  |  |  |  |  |  |  |  |  |
| Stefan Stender | 1183/34671 | 2923/43008 |  |  |  |  | 536/13889 | 3570/63790 |  |  |  |  | 1508/36515 | 2598/41164 |
| Sang-Wook Yi |  |  |  |  |  |  |  |  |  |  |  |  |  |  |
| Yen-Chun Chen | 35/425 | 106/1296 | 28/543 | 113/1178 |  |  |  |  |  |  |  |  |  |  |
| Bolanle O Ibitoye |  |  |  |  |  |  |  |  |  |  |  |  |  |  |
| Jung-Hwan Kim |  |  |  |  | 10/139 | 3/73 |  |  |  |  |  |  |  |  |
| I-Ching Lin | 410/6663 | 324/5251 |  |  |  |  |  |  |  |  |  |  |  |  |
| Chao Shen | 346/3699 | 195/1973 |  |  |  |  |  |  |  |  |  |  |  |  |
| Hsi-Che Shen | 484/3971 | 376/2540 |  |  |  |  |  |  | 335/3191 |  |  | 525/3320 |  |  |
| Yu Takahashi | 409/8625 | 289/2926 |  |  | 331/7226 | 363/8325 |  |  |  |  |  |  |  |  |
| Chung-Hung Tsai | 239/4726 | 208/3462 |  |  | 67/1312 | 380/6876 | 59/1293 | 388/6895 |  |  |  |  | 235/4090 | 212/4098 |
| Hong Yang |  |  |  |  |  |  |  |  |  |  |  |  |  |  |
| Farhad Zamani | 25/3462 | 26/2617 |  |  |  |  |  |  | 11/2062 |  |  | 40/4017 |  |  |
| Li Zhu | 768/5962 | 472/3493 |  |  |  |  |  |  |  |  |  |  |  |  |
| Alireza Ansari-Moghaddam | 11/796 | 29/726 |  |  |  |  |  |  |  | 26/620 |  | 14/900 | 6/509 | 34/1004 |
| Bo Kobberø Lauridsen | 1127/30101 | 2759/37725 |  |  | 1049/16229 | 2837/51597 | 505/12014 | 3381/55812 |  |  |  |  | 1438/32138 | 2448/35688 |
| Jun Lv | 7436/199292 | 20909/288081 |  |  | 4572/130243 | 23773/357130 | 2480/73706 | 25865/413667 |  |  |  |  |  |  |
| Amir Reza Radmard | 88/768 | 178/726 |  |  |  |  |  |  |  |  |  |  |  |  |
| Madhusudan Saha | 14/316 | 47/703 | 50/893 | 11/126 | 3/103 | 58/916 |  |  | 7/93 | 32/585 | 17/278 | 5/62 |  |  |
| Fen-Ming Zhang | 396/4352 | 486/5664 |  |  | 475/3496 | 407/6520 | 545/5008 | 337/5008 |  |  |  |  |  |  |
| Kazuo Inui | 561/13986 | 276/7564 |  |  |  |  |  |  |  |  |  |  |  |  |
| Yasir Mehmood |  |  |  |  |  |  |  |  |  |  |  |  |  |  |
| Seungho Ryu |  | 3341/83275 |  |  |  |  |  |  |  |  |  |  |  |  |
| Seungho Ryu | 4765/217403 | 4652/179317 |  |  | 2288/107511 | 7129/289209 | 1488/65062 | 7929/331658 |  |  |  |  | 1657/63078 | 7760/333642 |
| Chang Hee Kwon | 1106/37557 | 320/9336 |  |  | 384/12280 | 1042/34611 |  |  | 733/28706 |  |  | 693/18187 | 200/6855 | 1226/40038 |
| Oh-Sung Kwon | 451/11066 | 370/9697 | 206/7803 | 615/12139 |  |  |  |  | 10/393 | 163/5293 | 168/4317 | 468/10308 |  |  |
| Tong Liu | 3272/70172 | 1057/18775 |  |  |  |  |  |  |  |  |  |  |  |  |
| Faiza Qayyum | 1782/44612 | 4175/55537 |  |  | 1438/20984 | 4519/79165 | 1007/21745 | 4950/78404 |  |  |  |  | 2305/49263 | 3652/50886 |
| Mei-Yan Xu | 606/12073 | 389/5898 |  | 608/4453 |  |  |  |  | 24/569 | 337/7517 | 356/5565 | 148/1914 |  |  |
| Shengguang Yan | 102/2689 | 327/2761 |  |  |  |  |  |  |  |  |  |  |  |  |
| Yan Zheng |  |  |  |  | 2417/29037 | 6101/100942 | 5449/92653 | 3069/37326 |  |  |  |  |  |  |
| Hsin-Yin Hsu | 675/8146 | 473/7525 |  |  |  |  |  |  | 876/13101 |  |  | 272/2570 | 305/3938 | 843/11723 |
| Bukunmi Michael Idowu |  |  | 11/656 |  |  |  |  |  |  |  |  |  |  |  |
| Sathiyamoorthy Jothy |  |  | 16/600 |  |  |  |  |  |  |  |  |  |  |  |
| Hyung Sun Kim |  |  |  |  |  |  |  |  |  |  |  |  |  |  |
| Fei-Lin Su | 601/8678 | 439/5877 |  |  | 84/1590 | 926/12965 | 102/1898 | 938/12657 |  |  |  |  |  |  |
| Pei-Yuan Su |  |  | 50/1205 | 157/1589 |  |  |  |  |  |  |  |  |  |  |
| Heather A Ward | 3917/100255 | 19836/234731 |  |  | 4443/71299 | 18958/258074 | 19484/246349 | 4249/88832 |  |  |  |  | 7323/78002 | 16430/256984 |
| Qiyun Gu | 56/1237 | 38/1051 |  |  |  |  |  |  |  |  |  |  |  |  |
| Dan Huang | 8548/345775 | 8619/358662 |  |  |  |  |  |  |  |  |  |  |  |  |
| Sen Tao Song | 109/1753 | 165/2256 |  |  |  |  |  |  |  |  |  |  |  |  |
| Jiwen Wang | 102492/1133945 | 65600/934578 |  |  |  |  |  |  | 3382/96669 | 68131/976542 | 91413/935028 |  |  |  |
| Kazuya Higashizono | 11046/263946 | 12797/347988 | 661/50187 | 23182/561743 | 2392/72363 | 21451/539567 | 11665/302989 | 12178/308941 |  |  |  |  |  |  |
| Zeinab Sadri | 37/1557 | 142/2068 |  |  | 24/884 | 148/2737 | 5/229 | 167/3392 |  |  |  |  |  |  |
| Yang Song | 259739/5838763 | 257255/5099230 | 278090/7928845 | 238904/3009148 |  |  |  |  | 12151/464574 | 231299/5541018 | 170951/3006047 | 34132/545393 |  |  |
| Hoyoung Wang | 102/1784 | 76/1254 |  |  | 43/658 | 135/2380 | 105/1806 | 73/1232 |  |  |  |  |  |  |
| Jin Wang | 139/2309 | 395/2430 |  |  | 257/2035 | 277/2758 | 21/317 | 20/364 |  |  |  |  | 379/3635 | 155/1158 |
| Xinhe Zhang | 3459/155250 | 3078/128879 | 3208/211725 | 3329/72404 |  |  |  |  |  |  |  |  |  |  |
| Yalan Zhang | 12122/171276 | 15341/147127 | 11642/211308 | 15815/107047 |  |  |  |  | 301/11525 | 9681/151740 | 12137/115043 | 4937/35826 |  |  |
| Tien-Shin Chou | 82/788 | 117/1475 | 33/658 | 166/1605 |  |  | 88/1009 | 110/1244 |  |  |  |  | 99/1196 | 99/1059 |
| Signe E J Hansen | 1218/48720 | 3225/59718 |  |  |  |  |  |  |  |  |  |  |  |  |
| Li Jiang | 101/1551 | 91/827 |  |  |  |  |  |  |  |  |  |  |  |  |
| Aynur Unalp-Arida | 376/4479 | 901/4753 |  |  | 595/3856 | 680/5376 | 662/5619 | 435/3613 |  |  |  |  |  |  |
| Santiago Henao-Morán |  | 609/4953 |  |  | 223/2000 | 386/2953 |  |  |  |  |  |  |  |  |
| Chan Nie | 1959/29198 | 3369/43695 |  |  | 1009/15310 | 4319/57583 | 654/10176 | 4674/62717 |  |  |  |  |  |  |
| Keng-Wei Liang | 349/7388 | 589/13291 |  |  |  |  |  |  |  |  |  |  |  |  |
| Sailimai Man |  |  |  |  |  |  |  |  |  |  |  |  |  |  |
| Shridhar Dwivedi |  |  |  |  |  |  |  |  |  |  |  |  |  |  |
| Wenxiang Li |  |  |  |  |  |  |  |  |  |  |  |  |  |  |
| Hugh Shunsuke Colvin | 1495/29715 | 1597/32618 |  |  | 816/16810 | 2276/45523 |  |  |  |  |  |  |  |  |
| Yuanjie Pang | 8096/210205 | 22687/302510 |  |  | 6041/150138 | 24742/35999 | 2714/76088 | 28069/434049 |  |  |  |  |  |  |
| Stella Nimanya | 37/178 | 75/333 | 74/398 | 35/111 |  |  | 31/92 | 81/419 | 1/9 | 47/236 | 26/125 | 29/123 | 61/275 | 51/236 |
| Adam Gyedu |  |  |  |  |  |  |  |  |  |  |  |  |  |  |
| AG Kebede | 24/784 | 44/816 |  |  |  |  |  |  |  |  |  |  |  |  |
| [Oh-Sung Kwon](https://pubmed.ncbi.nlm.nih.gov/?sort=date&size=200&term=Kwon+OS&cauthor_id=32590752) | 217/4696 | 177/4212 |  |  |  |  |  |  | 5/182 | 94/2485 | 98/2257 | 197/3984 | 72/1886 | 322/7022 |
| Xinyu Zhao |  |  |  |  | 691/33113 | 1226/44607 | 737/35008 | 1179/42743 |  |  |  |  | 151/7446 | 1709/69387 |
| Guochong Jia | 204/35989 | 839/48377 |  |  |  |  |  |  |  |  |  |  |  |  |
| Young-Kyu Kim | 201/4313 | 154/3573 | 75/2380 | 280/5506 |  |  |  |  | 4/149 | 80/2052 | 78/1850 | 177/3638 |  |  |
| Xu Li | 35/375 | 44/522 | 21/374 | 58/523 |  |  |  |  |  |  |  |  |  |  |
| Fei Wang | 891/7056 | 1707/9243 |  |  | 397/3096 | 2201/13203 | 477/3752 | 2121/12547 | 1029/88126 |  | 1569/8173 |  | 2323/14503 | 275/1796 |
| Jian-Han Chen |  |  |  |  |  |  |  |  |  |  |  |  |  |  |
| Daniel Mønsted Shabanzadeh | 209/3022 | 382/2906 |  |  | 307/3145 | 283/2782 |  |  |  |  |  |  | 432/4372 | 159/1556 |
| Sabina Waniek | 25/358 | 21/224 |  |  | 3/66 | 43/516 |  |  |  |  |  |  |  |  |
| Yen-Ling Liu |  |  |  |  |  |  |  |  |  |  |  |  |  |  |
| Qiao-Hua Qiao | 654/4335 | 265/2329 |  |  |  |  |  |  |  |  |  |  |  |  |
| Ibinabo Ibiebele |  |  |  |  | 430/142089 | 1452/922000 |  |  |  |  |  |  |  |  |
| J-Y Wang | 240/3713 | 167/2498 |  |  | 32/563 | 375/5648 | 33/634 | 374/5577 |  |  |  |  | 17/340 | 390/5871 |
| T J McConnell | 176/11592 | 1006/38060 |  |  | 159/5640 | 1023/44012 |  |  |  |  |  |  |  |  |
| Qian Zhu | 604/11611 | 269/6680 |  |  |  |  |  |  |  |  |  |  |  |  |
| Min-Sun Kwak | 560/8682 | 509/8930 |  |  | 135/2533 | 934/15079 |  |  |  |  |  |  | 773/11608 | 296/6004 |
| Jia Liu | 274/5227 | 224/5973 |  |  |  |  |  |  |  |  |  |  |  |  |
| Yen-Chun Lee | 436/7121 | 332/4912 | 82/3040 |  | 68/1328 | 700/10705 | 58/1339 | 710/10694 |  |  |  |  | 51/965 | 717/11068 |
| Jau-Yuan Chen | 8/621 | 15/675 | 7/782 | 16/514 |  |  |  |  |  |  |  |  |  |  |
| Zhao-Yan Jiang |  |  |  |  |  |  |  |  |  |  |  |  |  |  |
| Xian-Hua Liao |  |  |  |  |  |  |  |  |  |  |  |  |  |  |
| Chia-Yen Dai | 64/689 | 51/1012 | 29/784 | 86/917 |  |  |  |  |  |  |  |  |  |  |
| Hsueh-Chou Lai |  |  |  |  |  |  |  |  |  |  |  |  |  |  |
| Li-Ying Chen | 653/4978 | 265/2592 |  |  |  |  |  |  |  |  |  |  |  |  |
| Farrokh Seilanian Toosi | 2/207 | 13/392 | 8/182 | 7/417 |  |  |  |  | 9/157 |  | 6/442 |  |  |  |
| Kuan-Fu Liao |  |  |  |  |  |  |  |  |  |  |  |  |  |  |
| Paul J R Banim | 41/11133 | 94/13068 |  |  |  |  |  |  |  |  |  |  | 79/16940 | 56/7315 |
| I Halldestam | 20/261 | 22/240 |  |  |  |  |  |  |  |  |  |  |  |  |
| Kaewalin Rungsinaporn |  |  |  |  |  |  |  |  |  |  |  |  |  |  |
| E Corazziari | 777/15721 | 1015/13418 |  |  |  |  |  |  |  |  |  |  |  |  |
| Kristi L Storti |  | 1281/8010 |  |  | 64/472 |  |  |  |  |  |  | 283/1379 |  |  |
| Chung-Jyi Tsai | 2356/45912 |  |  |  |  |  |  |  |  |  |  |  |  |  |
| Edmund J Bini | 364/6339 | 598/7126 |  |  |  |  |  |  |  |  |  |  |  |  |
| S Kono | 174/7637 |  |  |  | 92/3513 | 82/3567 | 126/6223 | 48/1414 |  |  |  |  |  |  |
| Oleg V Reshetnikov | 18/817 | 89/861 |  |  |  |  |  |  |  |  |  |  |  |  |
| E S Schernhammer | 1799/48928 | 9696/104856 |  |  | 3182/34065 |  |  |  |  |  |  |  |  |  |

| **Study** | **Vegetarian** | | **Family history of GS** | | **Hypertension** | | **Diabetes mellitus** | | **MAFLD** | | **Helicobacter pylori infection** | |
| --- | --- | --- | --- | --- | --- | --- | --- | --- | --- | --- | --- | --- |
|  | **Yes** | **No** | **Yes** | **No** | **Yes** | **No** | **Yes** | **No** | **Yes** | **No** | **Yes** | **No** |
| Kratzer W |  |  |  |  |  |  |  |  |  |  |  |  |
| J A Simon |  |  |  |  |  |  |  |  |  |  |  |  |
| A P Brasca |  |  |  |  |  |  |  |  |  |  |  |  |
| Chapman BA |  |  |  |  |  |  |  |  |  |  |  |  |
| P L Moro |  |  |  |  |  |  |  |  |  |  |  |  |
| C E Ruhl |  |  |  |  |  |  |  |  |  |  |  |  |
| S C Dhar |  |  |  |  |  |  |  |  |  |  |  |  |
| [S Massarrat](https://pubmed.ncbi.nlm.nih.gov/?sort=date&size=200&term=Massarrat+S&cauthor_id=11350555) |  |  |  |  |  |  |  |  |  |  |  |  |
| V Singh |  |  |  |  |  |  |  |  |  |  |  |  |
| James E Everhart |  |  |  |  |  |  |  |  |  |  |  |  |
| T K Ferguson |  |  |  |  |  |  |  |  |  |  |  |  |
| Shih-Wei Lai |  |  |  |  |  |  |  |  | 20/385 | 52/643 |  |  |
| Masako Okamoto |  |  |  |  |  |  |  |  |  |  |  |  |
| G A Hossain |  |  |  |  |  |  |  |  |  |  |  |  |
| Kim-Choy Ng |  |  |  |  |  |  |  |  |  |  |  |  |
| G Salinas |  |  | 31/146 | 50/388 |  |  |  |  |  |  |  |  |
| Hidenari Sakuta |  |  |  |  |  |  |  |  |  |  |  |  |
| Henry Völzke |  |  |  |  |  |  |  |  |  |  |  |  |
| Chien-Hua Chen | 6/85 | 162/3248 | 6/57 | 162/3276 |  |  |  |  | 80/931 | 88/2402 |  |  |
| Volker Kaechele |  |  |  |  |  |  |  |  |  |  |  |  |
| Chi-Ming Liu |  |  |  |  |  |  |  |  |  |  |  |  |
| S A Abu-Eshy |  |  | 10/36 | 24/255 |  |  | 4/43 | 30/248 |  |  |  |  |
| Toshiharu Fujita |  |  |  |  |  |  |  |  |  |  |  |  |
| D Katsika |  |  |  |  |  |  |  |  |  |  |  |  |
| Andrea M Kriska |  |  |  |  |  |  | 320/1645 | 240/1498 |  |  |  |  |
| Yoosoo Chang |  |  |  |  |  |  |  |  |  |  |  |  |
| Hui Sun |  |  |  |  | 115/640 | 269/2933 |  |  |  |  |  |  |
| Thomas Walcher | 4/66 | 162/2053 | 53/428 | 100/1609 |  |  | 14/59 | 153/2064 |  |  |  |  |
| Davide Festi |  |  |  |  |  |  |  |  |  |  |  |  |
| W Kratzer |  |  |  |  |  |  |  |  |  |  |  |  |
| Gangwar Richa |  |  |  |  |  |  |  |  |  |  |  |  |
| Sang Soo Kim |  |  |  |  |  |  |  |  | 32/732 | 141/3393 |  |  |
| Constance E Ruhl |  |  |  |  |  |  |  |  |  |  |  |  |
| Eric N Taylor |  |  |  |  | 3511/32633 | 11158/214622 | 660/4781 | 14009/242474 |  |  |  |  |
| Sayeed Unisa |  |  |  |  |  |  |  |  |  |  |  |  |
| Corinna Koebnick |  |  |  |  |  |  |  |  |  |  |  |  |
| Qing Xu |  |  |  |  | 719/7703 | 1808/48393 | 241/2486 | 2286/53610 | 1245/21468 | 1282/34628 |  |  |
| Hua-Li Yang |  |  |  |  |  |  |  |  |  |  |  |  |
| Xin Huang |  |  |  |  |  |  |  |  |  |  |  |  |
| Yu-Shan Mao |  |  |  |  |  |  |  |  |  |  |  |  |
| Stefan Stender |  |  |  |  |  |  |  |  |  |  |  |  |
| Sang-Wook Yi |  |  |  |  |  |  |  |  |  |  |  |  |
| Yen-Chun Chen |  |  |  |  |  |  |  |  |  |  |  |  |
| Bolanle O Ibitoye |  |  |  |  |  |  |  |  |  |  |  |  |
| Jung-Hwan Kim |  |  |  |  | 3/27 | 10/186 | 1/12 | 12/201 |  |  |  |  |
| I-Ching Lin |  |  |  |  | 412/5120 | 321/6778 |  |  |  |  |  |  |
| Chao Shen |  |  |  |  |  |  |  |  |  |  |  |  |
| Hsi-Che Shen |  |  |  |  | 329/2027 | 531/4484 |  |  |  |  |  |  |
| Yu Takahashi |  |  |  |  |  |  |  |  |  |  | 273/4493 | 421/11058 |
| Chung-Hung Tsai |  |  |  |  |  |  |  |  |  |  |  |  |
| Hong Yang |  |  |  |  |  |  |  |  |  |  |  |  |
| Farhad Zamani |  |  |  |  |  |  |  |  | 1/402 | 50/5677 |  |  |
| Li Zhu |  |  |  |  | 416/2408 | 824/7047 | 96/400 | 1144/9055 | 572/3378 | 668/6077 |  |  |
| Alireza Ansari-Moghaddam |  |  |  |  |  |  |  |  | 33/617 | 17/903 |  |  |
| Bo Kobberø Lauridsen |  |  |  |  | 2448/38289 | 1438/29537 | 194/2481 | 3692/65345 |  |  |  |  |
| Jun Lv |  |  |  |  | 9110/164475 | 19235/322898 | 1863/26150 | 26482/461223 |  |  |  |  |
| Amir Reza Radmard |  |  |  |  |  |  |  |  | 149/914 | 117/580 |  |  |
| Madhusudan Saha |  |  |  |  |  |  | 3/63 | 58/956 |  |  |  |  |
| Fen-Ming Zhang |  |  |  |  |  |  |  |  | 572/4383 | 310/5633 | 323/3410 | 559/6606 |
| Kazuo Inui |  |  |  |  |  |  |  |  |  |  |  |  |
| Yasir Mehmood |  |  |  |  |  |  |  |  |  |  |  |  |
| Seungho Ryu |  |  |  |  |  |  |  |  |  |  |  |  |
| Seungho Ryu |  |  |  |  |  |  | 509/10315 | 8908/29355 |  |  |  |  |
| Chang Hee Kwon |  |  |  |  | 295/7470 | 1131/39423 | 119/2327 | 1307/44566 | 672/19277 | 754/27616 |  |  |
| Oh-Sung Kwon |  |  |  |  |  |  |  |  |  |  |  |  |
| Tong Liu |  |  |  |  |  |  |  |  |  |  |  |  |
| Faiza Qayyum |  |  |  |  | 1470/13517 | 4487/86632 | 494/4385 | 5463/95764 |  |  |  |  |
| Mei-Yan Xu |  |  |  |  | 482/4203 | 513/13768 | 247/1951 | 748/16020 | 481/6138 | 514/11833 | 432/7803 | 503/10168 |
| Shengguang Yan |  |  |  |  |  |  |  |  |  |  |  |  |
| Yan Zheng |  |  |  |  | 2080/22426 | 6438/107553 |  |  |  |  |  |  |
| Hsin-Yin Hsu |  |  |  |  | 286/2280 | 862/13391 | 117/854 | 1031/14817 |  |  |  |  |
| Bukunmi Michael Idowu |  |  |  |  |  |  |  |  |  |  |  |  |
| Sathiyamoorthy Jothy |  |  |  |  |  |  |  |  |  |  |  |  |
| Hyung Sun Kim |  |  |  |  |  |  |  |  |  |  |  |  |
| Fei-Lin Su |  |  |  |  | 317/2824 | 723/11731 | 246/2092 | 794/12463 | 474/5443 | 566/9112 |  |  |
| Pei-Yuan Su |  |  |  |  |  |  |  |  |  |  |  |  |
| Heather A Ward |  |  |  |  |  |  | 1745/9911 | 22008/325075 |  |  |  |  |
| Qiyun Gu |  |  |  |  |  |  |  |  |  |  |  |  |
| Dan Huang |  |  |  |  |  |  | 3376/71440 | 13791/632997 | 1193/23637 | 15974/680800 |  |  |
| Sen Tao Song |  |  |  |  |  |  |  |  |  |  |  |  |
| Jiwen Wang |  |  |  |  |  |  |  |  |  |  |  |  |
| Kazuya Higashizono |  |  |  |  | 13425/278464 | 10418/333466 | 1555/28533 | 22288/583397 |  |  |  |  |
| Zeinab Sadri |  |  |  |  |  |  | 46/458 | 127/3167 | 78/755 | 95/1870 |  |  |
| Yang Song |  |  |  |  |  |  |  |  |  |  |  |  |
| Hoyoung Wang |  |  |  |  | 86/1063 | 92/1975 | 45/411 | 133/2627 |  |  |  |  |
| Jin Wang |  |  |  |  | 265/1290 | 269/2969 | 122/551 | 412/4242 |  |  |  |  |
| Xinhe Zhang |  |  |  |  |  |  |  |  |  |  |  |  |
| Yalan Zhang |  |  |  |  | 9048/69372 | 18415/249031 |  |  |  |  |  |  |
| Tien-Shin Chou |  |  |  |  |  |  |  |  |  |  |  |  |
| Signe E J Hansen |  |  |  |  |  |  |  |  |  |  |  |  |
| Li Jiang |  |  |  |  |  |  |  |  |  |  |  |  |
| Aynur Unalp-Arida |  |  |  |  | 625/2857 | 652/6375 | 330/1420 | 947/7812 |  |  |  |  |
| Santiago Henao-Morán |  |  |  |  |  |  | 89/415 | 520/4538 |  |  |  |  |
| Chan Nie |  |  |  |  |  |  |  |  |  |  |  |  |
| Keng-Wei Liang |  |  |  |  |  |  |  |  |  |  |  |  |
| Sailimai Man |  |  |  |  |  |  |  |  |  |  |  |  |
| Shridhar Dwivedi |  |  |  |  |  |  |  |  |  |  |  |  |
| Wenxiang Li |  |  |  |  |  |  |  |  |  |  |  |  |
| Hugh Shunsuke Colvin |  |  |  |  |  |  | 182/2679 | 2910/69654 |  |  |  |  |
| Yuanjie Pang |  |  |  |  | 4340/59106 | 26443/451031 | 2247/29690 | 28536/480447 |  |  |  |  |
| Stella Nimanya |  |  |  |  |  |  | 22/80 | 87/390 |  |  |  |  |
| Adam Gyedu |  |  |  |  |  |  |  |  |  |  |  |  |
| AG Kebede |  |  |  |  |  |  |  |  |  |  |  |  |
| [Oh-Sung Kwon](https://pubmed.ncbi.nlm.nih.gov/?sort=date&size=200&term=Kwon+OS&cauthor_id=32590752) |  |  |  |  |  |  |  |  | 205/3827 | 189/5081 |  |  |
| Xinyu Zhao |  |  |  |  |  |  | 282/7761 | 1723/72048 |  |  |  |  |
| Guochong Jia |  |  |  |  |  |  |  |  |  |  |  |  |
| Young-Kyu Kim |  |  |  |  |  |  |  |  |  |  |  |  |
| Xu Li |  |  |  |  | 27/216 | 52/681 | 24/171 | 55/726 | 43/367 | 36/530 |  |  |
| Fei Wang |  |  |  |  | 1377/7597 | 1221/8702 |  |  |  |  |  |  |
| Jian-Han Chen |  |  |  |  |  |  |  |  |  |  |  |  |
| Daniel Mønsted Shabanzadeh |  |  |  |  |  |  |  |  |  |  |  |  |
| Sabina Waniek |  |  |  |  | 34/400 | 12/182 | 7/61 | 39/521 |  |  |  |  |
| Yen-Ling Liu |  |  |  |  |  |  |  |  |  |  |  |  |
| Qiao-Hua Qiao |  |  |  |  | 187/1037 | 732/6816 | 51/243 | 868/7610 | 542/3646 | 377/4207 |  |  |
| Ibinabo Ibiebele |  |  |  |  | 254/94389 | 1628/969700 | 152/70226 | 1730/993863 |  |  |  |  |
| J-Y Wang |  |  |  |  |  |  |  |  |  |  |  |  |
| T J McConnell | 317/16176 | 865/33476 |  |  |  |  |  |  |  |  |  |  |
| Qian Zhu |  |  |  |  |  |  |  |  |  |  |  |  |
| Min-Sun Kwak |  |  |  |  | 311/3021 | 758/14591 | 100/871 | 969/16741 | 441/5337 | 628/12275 |  |  |
| Jia Liu |  |  |  |  |  |  |  |  | 289/4713 | 209/6487 |  |  |
| Yen-Chun Lee |  |  |  |  | 225/2292 | 543/9741 | 151/1425 | 617/10608 | 352/4475 | 416/7558 |  |  |
| Jau-Yuan Chen |  |  |  |  |  |  |  |  | 17/689 | 6/607 |  |  |
| Zhao-Yan Jiang |  |  |  |  |  |  |  |  |  |  |  |  |
| Xian-Hua Liao |  |  |  |  |  |  |  |  | 97/677 | 199/1928 |  |  |
| Chia-Yen Dai |  |  |  |  |  |  |  |  | 69/945 | 46/756 |  |  |
| Hsueh-Chou Lai |  |  |  |  |  |  | 3808/214179 | 2315/206860 |  |  |  |  |
| Li-Ying Chen |  |  |  |  |  |  |  |  |  |  |  |  |
| Farrokh Seilanian Toosi |  |  |  |  |  |  | 4/12 | 11/587 |  |  |  |  |
| Kuan-Fu Liao |  |  |  |  |  |  | 3145/49803 | 9637/199212 |  |  |  |  |
| Paul J R Banim |  |  |  |  |  |  |  |  |  |  |  |  |
| I Halldestam |  |  |  |  |  |  |  |  |  |  |  |  |
| Kaewalin Rungsinaporn |  |  |  |  |  |  |  |  |  |  |  |  |
| E Corazziari |  |  |  |  |  |  |  |  |  |  |  |  |
| Kristi L Storti |  |  |  |  |  |  | 141/523 | 1140/7487 |  |  |  |  |
| Chung-Jyi Tsai |  |  |  |  |  |  |  |  |  |  |  |  |
| Edmund J Bini |  |  |  |  |  |  |  |  |  |  |  |  |
| S Kono |  |  |  |  |  |  |  |  |  |  |  |  |
| Oleg V Reshetnikov |  |  |  |  |  |  |  |  |  |  |  |  |
| E S Schernhammer |  |  |  |  |  |  | 727/4662 | 10768/149122 |  |  |  |  |

| author | case | event-normal TC | case | event-high TC | case | event-normal TG | case | TG | case | normal HDL | case | low HDL | case | normal LDL | case | high LDL |
| --- | --- | --- | --- | --- | --- | --- | --- | --- | --- | --- | --- | --- | --- | --- | --- | --- |
| Chien-Hua Chen | 83 | 1942 | 82 | 1317 | 111 | 2444 | 54 | 815 |  |  |  |  |  |  |  |  |
| Chi-Ming Liu | 93 | 1920 | 33 | 466 | 100 | 2046 | 26 | 340 | 122 | 2174 | 4 | 212 |  |  |  |  |
| Hui Sun | 343 | 3323 | 41 | 250 | 206 | 2445 | 178 | 1128 | 358 | 3440 | 26 | 133 | 360 | 3429 | 24 | 144 |
| Thomas Walcher | 107 | 1418 | 55 | 686 | 120 | 1544 | 28 | 390 | 125 | 1756 | 32 | 295 | 118 | 1440 | 21 | 333 |
| Qing Xu | 2229 | 51043 | 298 | 5053 | 2037 | 46553 | 490 | 9543 | 2357 | 53188 | 170 | 2908 | 2205 | 50454 | 322 | 5642 |
| I-Ching Lin |  |  |  |  | 538 | 9193 | 184 | 2551 | 374 | 6920 | 316 | 3826 |  |  |  |  |
| Hsi-Che Shen | 505 | 4154 | 355 | 2357 | 542 | 4236 | 318 | 2275 | 609 | 4083 | 251 | 2428 |  |  |  |  |
| Chung-Hung Tsai |  |  |  |  | 336 | 6334 | 111 | 1854 | 273 | 5432 | 174 | 2756 |  |  |  |  |
| Alireza Ansari-Moghaddam | 25 | 900 | 15 | 612 | 29 | 946 | 11 | 566 | 26 | 1189 | 14 | 333 | 14 | 569 | 26 | 943 |
| Oh-Sung Kwon | 472 | 11407 | 349 | 9356 | 606 | 15423 | 215 | 5332 | 667 | 17912 | 154 | 2843 | 505 | 12695 | 298 | 7610 |
| Mei-Yan Xu | 861 | 15695 | 134 | 2276 | 448 | 9291 | 574 | 8680 | 657 | 12715 | 338 | 5256 | 704 | 13147 | 291 | 4824 |
| Jiwen Wang | 98758 | 1285494 | 63628 | 661487 | 105758 | 1371651 | 56400 | 571265 | 78421 | 900060 | 21805 | 207201 | 69086 | 818909 | 30900 | 285862 |
| Yang Song |  |  |  |  | 415071 | 8793655 | 87654 | 1573226 |  |  |  |  |  |  |  |  |
| Yalan Zhang | 19899 | 238584 | 6557 | 63864 | 15420 | 204197 | 11035 | 98242 | 24313 | 277978 | 1046 | 9596 | 19614 | 231447 | 5690 | 55714 |
| Tien-Shin Chou | 83 | 927 | 116 | 1336 |  |  |  |  |  |  |  |  | 115 | 1316 | 84 | 947 |
| Li Jiang |  |  |  |  | 136 | 1693 | 56 | 685 | 147 | 2031 | 45 | 347 |  |  |  |  |
| Oh-Sung Kwon | 202 | 4327 | 173 | 3888 | 280 | 6289 | 97 | 1955 | 308 | 7171 | 69 | 1073 | 223 | 5063 | 157 | 3004 |
| Xinyu Zhao | 1561 | 78970 | 434 | 20640 | 1480 | 58040 | 512 | 21171 | 1435 | 58989 | 519 | 18743 |  |  |  |  |
| Young-Kyu Kim | 191 | 4172 | 164 | 3174 | 265 | 5994 | 90 | 1892 | 290 | 6854 | 65 | 1032 | 209 | 4773 | 136 | 2930 |

**Supplementary Table 3. Sensitivity analysis and leave-one-out analysis for including studies**

|  | resid | se | z |
| --- | --- | --- | --- |
| **101** | 0.2446 | 0.0891 | 2.7436 |
| **3** | 0.2263 | 0.0878 | 2.5783 |
| **4** | 0.2307 | 0.0912 | 2.5306 |
| **36** | -0.2078 | 0.0868 | -2.3936 |
| **115** | -0.2044 | 0.0869 | -2.3534 |
| **74** | 0.1961 | 0.087 | 2.2534 |
| **25** | 0.1923 | 0.0875 | 2.1965 |
| **57** | 0.1923 | 0.0881 | 2.1828 |
| **39** | -0.1726 | 0.0874 | -1.9744 |
| **130** | -0.1712 | 0.0875 | -1.9572 |
| **134** | 0.1677 | 0.0876 | 1.9135 |
| **10** | 0.1681 | 0.0879 | 1.9129 |
| **109** | 0.1669 | 0.0876 | 1.9068 |
| **123** | 0.1679 | 0.0886 | 1.8943 |
| **52** | -0.1539 | 0.0879 | -1.7516 |
| **16** | 0.157 | 0.0903 | 1.7386 |
| **5** | 0.1467 | 0.0887 | 1.6535 |
| **110** | -0.1382 | 0.088 | -1.57 |
| **92** | 0.1371 | 0.088 | 1.5581 |
| **106** | -0.1345 | 0.0879 | -1.5304 |
| **30** | -0.1394 | 0.0925 | -1.5075 |
| **6** | 0.1307 | 0.0882 | 1.4819 |
| **48** | 0.128 | 0.0882 | 1.4511 |
| **53** | 0.1265 | 0.0881 | 1.4359 |
| **126** | -0.125 | 0.088 | -1.4206 |
| **97** | -0.1346 | 0.0949 | -1.4194 |
| **93** | 0.1142 | 0.0884 | 1.292 |
| **127** | 0.1116 | 0.0883 | 1.2633 |
| **72** | -0.1131 | 0.0903 | -1.2529 |
| **122** | -0.1108 | 0.0892 | -1.2413 |
| **114** | 0.105 | 0.0884 | 1.1879 |
| **22** | 0.1066 | 0.093 | 1.1464 |
| **23** | -0.101 | 0.0883 | -1.1438 |
| **124** | 0.0998 | 0.0888 | 1.1238 |
| **96** | -0.0983 | 0.0883 | -1.1138 |
| **86** | 0.0982 | 0.0886 | 1.1086 |
| **20** | -0.0994 | 0.0911 | -1.0912 |
| **26** | -0.0948 | 0.0884 | -1.0733 |
| **137** | -0.0939 | 0.0885 | -1.0618 |
| **87** | -0.0934 | 0.0883 | -1.0578 |
| **51** | -0.0925 | 0.0884 | -1.0464 |
| **98** | -0.091 | 0.0883 | -1.0308 |
| **63** | -0.091 | 0.0883 | -1.0301 |
| **117** | -0.0907 | 0.0883 | -1.0269 |
| **27** | 0.0898 | 0.0887 | 1.0126 |
| **79** | -0.0889 | 0.0883 | -1.0064 |
| **61** | -0.0921 | 0.0918 | -1.0038 |
| **18** | 0.0876 | 0.0888 | 0.9871 |
| **105** | -0.0864 | 0.0884 | -0.9782 |
| **128** | -0.0842 | 0.0907 | -0.928 |
| **54** | -0.0818 | 0.0893 | -0.9157 |
| **73** | -0.0791 | 0.0907 | -0.8718 |
| **111** | 0.0769 | 0.0887 | 0.8676 |
| **24** | -0.0758 | 0.0884 | -0.8572 |
| **44** | -0.0738 | 0.0895 | -0.8241 |
| **64** | -0.0702 | 0.0885 | -0.7933 |
| **47** | 0.0696 | 0.0887 | 0.7846 |
| **9** | -0.0681 | 0.089 | -0.7654 |
| **59** | 0.0568 | 0.0887 | 0.6403 |
| **108** | 0.0575 | 0.0901 | 0.6385 |
| **89** | 0.0568 | 0.0892 | 0.6371 |
| **88** | 0.0535 | 0.0885 | 0.6039 |
| **1** | -0.0535 | 0.0893 | -0.5987 |
| **38** | -0.0523 | 0.0887 | -0.5902 |
| **131** | 0.0506 | 0.0913 | 0.5539 |
| **60** | -0.0469 | 0.0886 | -0.5292 |
| **82** | -0.0466 | 0.0886 | -0.5265 |
| **43** | 0.0461 | 0.0894 | 0.5153 |
| **65** | -0.0451 | 0.0886 | -0.5086 |
| **8** | -0.0451 | 0.0893 | -0.5047 |
| **81** | 0.0444 | 0.0886 | 0.5012 |
| **14** | 0.0442 | 0.0896 | 0.493 |
| **21** | -0.0439 | 0.0892 | -0.4927 |
| **62** | -0.0436 | 0.0886 | -0.4925 |
| **91** | 0.0438 | 0.0892 | 0.4907 |
| **90** | -0.0415 | 0.0886 | -0.468 |
| **17** | -0.0417 | 0.09 | -0.4629 |
| **112** | 0.0416 | 0.091 | 0.4573 |
| **78** | -0.0407 | 0.0892 | -0.456 |
| **35** | -0.0399 | 0.0888 | -0.4488 |
| **69** | 0.0398 | 0.0889 | 0.4481 |
| **132** | -0.0398 | 0.089 | -0.4472 |
| **28** | 0.0395 | 0.0893 | 0.4428 |
| **37** | -0.0387 | 0.0886 | -0.4364 |
| **32** | -0.0388 | 0.0889 | -0.4357 |
| **103** | -0.037 | 0.0895 | -0.414 |
| **11** | -0.037 | 0.091 | -0.4063 |
| **29** | 0.0351 | 0.0887 | 0.3951 |
| **113** | 0.0347 | 0.0888 | 0.3902 |
| **95** | -0.0336 | 0.0887 | -0.3789 |
| **104** | -0.0332 | 0.0888 | -0.3742 |
| **120** | -0.0327 | 0.0887 | -0.3682 |
| **49** | -0.0323 | 0.0887 | -0.3642 |
| **139** | 0.0322 | 0.0886 | 0.3629 |
| **107** | -0.0313 | 0.0888 | -0.3524 |
| **71** | 0.0293 | 0.0887 | 0.3308 |
| **94** | 0.029 | 0.0887 | 0.327 |
| **31** | -0.0285 | 0.0906 | -0.3145 |
| **84** | -0.026 | 0.0886 | -0.2937 |
| **75** | 0.0258 | 0.0887 | 0.2912 |
| **136** | 0.0258 | 0.0887 | 0.2911 |
| **33** | 0.0249 | 0.0887 | 0.281 |
| **118** | -0.0249 | 0.0887 | -0.2804 |
| **77** | 0.0247 | 0.0886 | 0.2789 |
| **83** | -0.0247 | 0.089 | -0.2772 |
| **2** | 0.0243 | 0.0888 | 0.2742 |
| **12** | 0.0238 | 0.09 | 0.2647 |
| **13** | -0.0229 | 0.0888 | -0.2576 |
| **66** | -0.0227 | 0.0887 | -0.2562 |
| **99** | -0.0205 | 0.0887 | -0.2315 |
| **80** | 0.0199 | 0.089 | 0.2232 |
| **102** | -0.0194 | 0.0891 | -0.2179 |
| **125** | 0.0187 | 0.0895 | 0.2087 |
| **19** | -0.0184 | 0.0891 | -0.2064 |
| **45** | 0.0189 | 0.0928 | 0.2035 |
| **135** | -0.0166 | 0.0887 | -0.187 |
| **129** | -0.0166 | 0.0887 | -0.1869 |
| **40** | -0.0152 | 0.0888 | -0.1708 |
| **116** | 0.0141 | 0.0889 | 0.1588 |
| **70** | 0.014 | 0.0887 | 0.1577 |
| **41** | -0.0131 | 0.0887 | -0.1477 |
| **76** | 0.0129 | 0.0891 | 0.1453 |
| **138** | 0.0109 | 0.0895 | 0.1217 |
| **121** | 0.0106 | 0.0888 | 0.1189 |
| **7** | -0.0099 | 0.09 | -0.11 |
| **50** | -0.0091 | 0.0888 | -0.1026 |
| **68** | -0.0075 | 0.0887 | -0.0842 |
| **46** | 0.006 | 0.0888 | 0.067 |
| **133** | 0.0057 | 0.0887 | 0.064 |
| **119** | 0.004 | 0.0887 | 0.0452 |
| **55** | -0.0033 | 0.0887 | -0.0372 |
| **100** | 0.0032 | 0.0887 | 0.0362 |
| **58** | 0.0031 | 0.09 | 0.0345 |
| **42** | 0.0019 | 0.0887 | 0.0213 |
| **56** | -0.0014 | 0.0887 | -0.0163 |
| **67** | 0.0014 | 0.0887 | 0.0157 |
| **15** | 0.0013 | 0.0891 | 0.0143 |
| **34** | 0.0011 | 0.0887 | 0.012 |
| **85** | -0.0002 | 0.0891 | -0.0023 |

**Supplementary Table 4. Leave one out analysis by using a build-in function for including studies.**

|  | **rstudent** | **dffits** | **cook.d** | **cov.r** | **tau2.del** | **QE.del** | **hat** | **weight** | **dfbs** |
| --- | --- | --- | --- | --- | --- | --- | --- | --- | --- |
| **1** | -0.5987 | -0.051 | 0.0026 | 1.012 | 0.0078 | 359995.063 | 0.0072 | 0.7176 | -0.051 |
| **2** | 0.2742 | 0.0233 | 0.0005 | 1.0143 | 0.0078 | 359866.629 | 0.0073 | 0.7273 | 0.0233 |
| **3** | 2.5783 | 0.2188 | 0.046 | 0.9675 | 0.0074 | 359683.39 | 0.0071 | 0.7102 | 0.2189 |
| **4** | 2.5306 | 0.2069 | 0.0414 | 0.9712 | 0.0075 | 359909.513 | 0.0066 | 0.6626 | 0.2072 |
| **5** | 1.6535 | 0.1405 | 0.0195 | 0.9946 | 0.0076 | 359802.222 | 0.0071 | 0.7145 | 0.1405 |
| **6** | 1.4819 | 0.1269 | 0.016 | 0.9984 | 0.0077 | 359390.531 | 0.0073 | 0.7256 | 0.1269 |
| **7** | -0.11 | -0.0094 | 0.0001 | 1.0144 | 0.0078 | 359995.31 | 0.0071 | 0.7081 | -0.0094 |
| **8** | -0.5047 | -0.043 | 0.0019 | 1.0128 | 0.0078 | 359996.955 | 0.0072 | 0.7174 | -0.043 |
| **9** | -0.7654 | -0.0653 | 0.0043 | 1.0104 | 0.0078 | 359985.902 | 0.0072 | 0.7209 | -0.0653 |
| **10** | 1.9129 | 0.1637 | 0.0263 | 0.9878 | 0.0076 | 359464.807 | 0.0072 | 0.7227 | 0.1636 |
| **11** | -0.4063 | -0.034 | 0.0012 | 1.0129 | 0.0078 | 359997.792 | 0.0069 | 0.6908 | -0.034 |
| **12** | 0.2647 | 0.0222 | 0.0005 | 1.0139 | 0.0078 | 359984.077 | 0.0071 | 0.7075 | 0.0222 |
| **13** | -0.2576 | -0.0222 | 0.0005 | 1.0144 | 0.0078 | 359992.624 | 0.0073 | 0.7274 | -0.0222 |
| **14** | 0.493 | 0.0416 | 0.0017 | 1.0127 | 0.0078 | 359965.293 | 0.0071 | 0.7125 | 0.0416 |
| **15** | 0.0143 | 0.0011 | 0 | 1.0148 | 0.0078 | 359982.518 | 0.0072 | 0.7221 | 0.0011 |
| **16** | 1.7386 | 0.1449 | 0.0207 | 0.9928 | 0.0076 | 359920.565 | 0.0069 | 0.6882 | 0.145 |
| **17** | -0.4629 | -0.0392 | 0.0015 | 1.0129 | 0.0078 | 359997.604 | 0.0071 | 0.7061 | -0.0392 |
| **18** | 0.9871 | 0.0842 | 0.0071 | 1.0074 | 0.0077 | 359799.838 | 0.0072 | 0.7228 | 0.0842 |
| **19** | -0.2064 | -0.0178 | 0.0003 | 1.0145 | 0.0078 | 359994.452 | 0.0072 | 0.7227 | -0.0178 |
| **20** | -1.0912 | -0.0906 | 0.0082 | 1.0056 | 0.0077 | 359989.567 | 0.0068 | 0.6849 | -0.0906 |
| **21** | -0.4927 | -0.0421 | 0.0018 | 1.0129 | 0.0078 | 359996.945 | 0.0072 | 0.72 | -0.0421 |
| **22** | 1.1464 | 0.0933 | 0.0087 | 1.0045 | 0.0077 | 359974.957 | 0.0066 | 0.657 | 0.0933 |
| **23** | -1.1438 | -0.098 | 0.0096 | 1.0051 | 0.0077 | 359224.735 | 0.0073 | 0.7292 | -0.098 |
| **24** | -0.8572 | -0.0735 | 0.0054 | 1.0094 | 0.0078 | 359601.168 | 0.0073 | 0.7293 | -0.0735 |
| **25** | 2.1965 | 0.188 | 0.0344 | 0.9796 | 0.0075 | 359361.069 | 0.0072 | 0.7223 | 0.188 |
| **26** | -1.0733 | -0.0919 | 0.0084 | 1.0062 | 0.0077 | 359716.42 | 0.0073 | 0.7285 | -0.0919 |
| **27** | 1.0126 | 0.0864 | 0.0075 | 1.0071 | 0.0077 | 359780.471 | 0.0072 | 0.7232 | 0.0864 |
| **28** | 0.4428 | 0.0375 | 0.0014 | 1.0132 | 0.0078 | 359951.973 | 0.0072 | 0.7188 | 0.0375 |
| **29** | 0.3951 | 0.0337 | 0.0011 | 1.0137 | 0.0078 | 359784.343 | 0.0073 | 0.7276 | 0.0337 |
| **30** | -1.5075 | -0.1226 | 0.0149 | 0.9981 | 0.0077 | 359984.413 | 0.0066 | 0.6604 | -0.1227 |
| **31** | -0.3145 | -0.0265 | 0.0007 | 1.0135 | 0.0078 | 359997.713 | 0.007 | 0.6977 | -0.0265 |
| **32** | -0.4357 | -0.0373 | 0.0014 | 1.0134 | 0.0078 | 359997.494 | 0.0072 | 0.7241 | -0.0373 |
| **33** | 0.281 | 0.0239 | 0.0006 | 1.0143 | 0.0078 | 359800.475 | 0.0073 | 0.7281 | 0.0239 |
| **34** | 0.012 | 0.0009 | 0 | 1.0149 | 0.0078 | 358760.787 | 0.0073 | 0.7296 | 0.0009 |
| **35** | -0.4488 | -0.0385 | 0.0015 | 1.0133 | 0.0078 | 359997.027 | 0.0073 | 0.7261 | -0.0385 |
| **36** | -2.3936 | -0.2045 | 0.0404 | 0.9731 | 0.0075 | 298003.948 | 0.0073 | 0.7297 | -0.2045 |
| **37** | -0.4364 | -0.0375 | 0.0014 | 1.0135 | 0.0078 | 359993.382 | 0.0073 | 0.7293 | -0.0375 |
| **38** | -0.5902 | -0.0506 | 0.0026 | 1.0122 | 0.0078 | 359983.285 | 0.0073 | 0.7277 | -0.0506 |
| **39** | -1.9744 | -0.1688 | 0.0279 | 0.9861 | 0.0076 | 356269.928 | 0.0073 | 0.7293 | -0.1688 |
| **40** | -0.1708 | -0.0148 | 0.0002 | 1.0146 | 0.0078 | 359982.598 | 0.0073 | 0.7275 | -0.0148 |
| **41** | -0.1477 | -0.0128 | 0.0002 | 1.0147 | 0.0078 | 359858.817 | 0.0073 | 0.7294 | -0.0128 |
| **42** | 0.0213 | 0.0017 | 0 | 1.0149 | 0.0078 | 359401.199 | 0.0073 | 0.7295 | 0.0017 |
| **43** | 0.5153 | 0.0437 | 0.0019 | 1.0126 | 0.0078 | 359953.9 | 0.0072 | 0.7163 | 0.0437 |
| **44** | -0.8241 | -0.0698 | 0.0049 | 1.0095 | 0.0078 | 359989.939 | 0.0071 | 0.7118 | -0.0698 |
| **45** | 0.2035 | 0.0165 | 0.0003 | 1.0133 | 0.0078 | 359994.113 | 0.0066 | 0.6648 | 0.0165 |
| **46** | 0.067 | 0.0056 | 0 | 1.0148 | 0.0078 | 359921.426 | 0.0073 | 0.7278 | 0.0056 |
| **47** | 0.7846 | 0.067 | 0.0045 | 1.0102 | 0.0078 | 359756.06 | 0.0073 | 0.7256 | 0.067 |
| **48** | 1.4511 | 0.1243 | 0.0153 | 0.9991 | 0.0077 | 359321.218 | 0.0073 | 0.7261 | 0.1243 |
| **49** | -0.3642 | -0.0313 | 0.001 | 1.0139 | 0.0078 | 359997.549 | 0.0073 | 0.7282 | -0.0313 |
| **50** | -0.1026 | -0.0089 | 0.0001 | 1.0148 | 0.0078 | 359977.225 | 0.0073 | 0.7269 | -0.0089 |
| **51** | -1.0464 | -0.0896 | 0.008 | 1.0067 | 0.0077 | 359687.454 | 0.0073 | 0.7287 | -0.0896 |
| **52** | -1.7516 | -0.1495 | 0.022 | 0.9921 | 0.0076 | 359654.956 | 0.0073 | 0.7259 | -0.1495 |
| **53** | 1.4359 | 0.1231 | 0.015 | 0.9994 | 0.0077 | 359032.315 | 0.0073 | 0.7272 | 0.1231 |
| **54** | -0.9157 | -0.0777 | 0.006 | 1.0084 | 0.0078 | 359984.305 | 0.0071 | 0.7146 | -0.0777 |
| **55** | -0.0372 | -0.0034 | 0 | 1.0149 | 0.0078 | 359739.03 | 0.0073 | 0.7294 | -0.0034 |
| **56** | -0.0163 | -0.0016 | 0 | 1.0149 | 0.0078 | 357868.509 | 0.0073 | 0.7297 | -0.0016 |
| **57** | 2.1828 | 0.1857 | 0.0336 | 0.9802 | 0.0075 | 359695.163 | 0.0071 | 0.7143 | 0.1857 |
| **58** | 0.0345 | 0.0028 | 0 | 1.0144 | 0.0078 | 359992.165 | 0.0071 | 0.7073 | 0.0028 |
| **59** | 0.6403 | 0.0547 | 0.003 | 1.0117 | 0.0078 | 359669.711 | 0.0073 | 0.7274 | 0.0547 |
| **60** | -0.5292 | -0.0455 | 0.0021 | 1.0128 | 0.0078 | 359984.426 | 0.0073 | 0.7286 | -0.0455 |
| **61** | -1.0038 | -0.0828 | 0.0069 | 1.0068 | 0.0077 | 359992.47 | 0.0068 | 0.6759 | -0.0828 |
| **62** | -0.4925 | -0.0423 | 0.0018 | 1.0131 | 0.0078 | 359969.461 | 0.0073 | 0.7294 | -0.0423 |
| **63** | -1.0301 | -0.0883 | 0.0078 | 1.0069 | 0.0077 | 354893.703 | 0.0073 | 0.7297 | -0.0883 |
| **64** | -0.7933 | -0.0681 | 0.0046 | 1.0102 | 0.0078 | 359759.886 | 0.0073 | 0.7292 | -0.0681 |
| **65** | -0.5086 | -0.0437 | 0.0019 | 1.0129 | 0.0078 | 359988.39 | 0.0073 | 0.7286 | -0.0437 |
| **66** | -0.2562 | -0.0221 | 0.0005 | 1.0144 | 0.0078 | 359950.006 | 0.0073 | 0.7295 | -0.0221 |
| **67** | 0.0157 | 0.0012 | 0 | 1.0149 | 0.0078 | 359490.727 | 0.0073 | 0.7295 | 0.0012 |
| **68** | -0.0842 | -0.0074 | 0.0001 | 1.0148 | 0.0078 | 359946.528 | 0.0073 | 0.7284 | -0.0074 |
| **69** | 0.4481 | 0.0382 | 0.0015 | 1.0133 | 0.0078 | 359879.555 | 0.0073 | 0.7254 | 0.0382 |
| **70** | 0.1577 | 0.0134 | 0.0002 | 1.0147 | 0.0078 | 358792.83 | 0.0073 | 0.7295 | 0.0134 |
| **71** | 0.3308 | 0.0282 | 0.0008 | 1.014 | 0.0078 | 359746.894 | 0.0073 | 0.7282 | 0.0282 |
| **72** | -1.2529 | -0.1048 | 0.0109 | 1.003 | 0.0077 | 359981.738 | 0.007 | 0.6955 | -0.1048 |
| **73** | -0.8718 | -0.0728 | 0.0053 | 1.0087 | 0.0078 | 359993.063 | 0.0069 | 0.6925 | -0.0728 |
| **74** | 2.2534 | 0.1939 | 0.0365 | 0.9776 | 0.0075 | 327439.258 | 0.0073 | 0.7296 | 0.1939 |
| **75** | 0.2912 | 0.0248 | 0.0006 | 1.0142 | 0.0078 | 359789.681 | 0.0073 | 0.7281 | 0.0248 |
| **76** | 0.1453 | 0.0122 | 0.0002 | 1.0146 | 0.0078 | 359969.654 | 0.0072 | 0.7224 | 0.0122 |
| **77** | 0.2789 | 0.0238 | 0.0006 | 1.0143 | 0.0078 | 355316.415 | 0.0073 | 0.7297 | 0.0238 |
| **78** | -0.456 | -0.0389 | 0.0015 | 1.0132 | 0.0078 | 359997.447 | 0.0072 | 0.7196 | -0.0389 |
| **79** | -1.0064 | -0.0863 | 0.0074 | 1.0073 | 0.0077 | 351465.605 | 0.0073 | 0.7297 | -0.0863 |
| **80** | 0.2232 | 0.0189 | 0.0004 | 1.0144 | 0.0078 | 359951.323 | 0.0072 | 0.7239 | 0.0189 |
| **81** | 0.5012 | 0.0428 | 0.0018 | 1.013 | 0.0078 | 304130.017 | 0.0073 | 0.7297 | 0.0429 |
| **82** | -0.5265 | -0.0453 | 0.0021 | 1.0128 | 0.0078 | 359623.815 | 0.0073 | 0.7297 | -0.0453 |
| **83** | -0.2772 | -0.0238 | 0.0006 | 1.0142 | 0.0078 | 359996.468 | 0.0072 | 0.7233 | -0.0238 |
| **84** | -0.2937 | -0.0253 | 0.0006 | 1.0143 | 0.0078 | 354008.505 | 0.0073 | 0.7297 | -0.0253 |
| **85** | -0.0023 | -0.0004 | 0 | 1.0148 | 0.0078 | 359983.832 | 0.0072 | 0.7221 | -0.0004 |
| **86** | 1.1086 | 0.0948 | 0.009 | 1.0056 | 0.0077 | 359669.551 | 0.0072 | 0.7248 | 0.0948 |
| **87** | -1.0578 | -0.0907 | 0.0082 | 1.0065 | 0.0077 | 356042.173 | 0.0073 | 0.7296 | -0.0907 |
| **88** | 0.6039 | 0.0517 | 0.0027 | 1.0121 | 0.0078 | 350170.618 | 0.0073 | 0.7297 | 0.0517 |
| **89** | 0.6371 | 0.0541 | 0.0029 | 1.0116 | 0.0078 | 359923.64 | 0.0072 | 0.7195 | 0.0541 |
| **90** | -0.468 | -0.0402 | 0.0016 | 1.0133 | 0.0078 | 359976.146 | 0.0073 | 0.7295 | -0.0402 |
| **91** | 0.4907 | 0.0417 | 0.0017 | 1.0129 | 0.0078 | 359940.577 | 0.0072 | 0.72 | 0.0417 |
| **92** | 1.5581 | 0.1336 | 0.0177 | 0.9967 | 0.0077 | 358926.99 | 0.0073 | 0.7272 | 0.1336 |
| **93** | 1.292 | 0.1105 | 0.0122 | 1.0023 | 0.0077 | 359566.656 | 0.0073 | 0.725 | 0.1105 |
| **94** | 0.327 | 0.0279 | 0.0008 | 1.0141 | 0.0078 | 358840.713 | 0.0073 | 0.7294 | 0.0279 |
| **95** | -0.3789 | -0.0326 | 0.0011 | 1.0138 | 0.0078 | 359997.76 | 0.0073 | 0.7286 | -0.0326 |
| **96** | -1.1138 | -0.0954 | 0.0091 | 1.0056 | 0.0077 | 359045.992 | 0.0073 | 0.7293 | -0.0954 |
| **97** | -1.4194 | -0.1127 | 0.0126 | 0.9999 | 0.0077 | 359989.838 | 0.0063 | 0.6285 | -0.1127 |
| **98** | -1.0308 | -0.0884 | 0.0078 | 1.0069 | 0.0077 | 356318.544 | 0.0073 | 0.7296 | -0.0884 |
| **99** | -0.2315 | -0.02 | 0.0004 | 1.0145 | 0.0078 | 359950.633 | 0.0073 | 0.7294 | -0.02 |
| **100** | 0.0362 | 0.0029 | 0 | 1.0149 | 0.0078 | 357090.432 | 0.0073 | 0.7297 | 0.0029 |
| **101** | 2.7436 | 0.2286 | 0.0501 | 0.9629 | 0.0074 | 359840.83 | 0.0069 | 0.6864 | 0.2289 |
| **102** | -0.2179 | -0.0187 | 0.0004 | 1.0144 | 0.0078 | 359995.323 | 0.0072 | 0.7215 | -0.0187 |
| **103** | -0.414 | -0.0353 | 0.0013 | 1.0134 | 0.0078 | 359997.762 | 0.0072 | 0.7153 | -0.0353 |
| **104** | -0.3742 | -0.0322 | 0.001 | 1.0138 | 0.0078 | 359997.763 | 0.0073 | 0.7271 | -0.0322 |
| **105** | -0.9782 | -0.0839 | 0.007 | 1.0077 | 0.0077 | 359141.575 | 0.0073 | 0.7294 | -0.0839 |
| **106** | -1.5304 | -0.131 | 0.017 | 0.9974 | 0.0077 | 356647.854 | 0.0073 | 0.7294 | -0.131 |
| **107** | -0.3524 | -0.0303 | 0.0009 | 1.0139 | 0.0078 | 359997.515 | 0.0073 | 0.7268 | -0.0303 |
| **108** | 0.6385 | 0.0537 | 0.0029 | 1.0114 | 0.0078 | 359967.929 | 0.007 | 0.7044 | 0.0537 |
| **109** | 1.9068 | 0.1638 | 0.0263 | 0.9879 | 0.0076 | 357392.227 | 0.0073 | 0.7283 | 0.1638 |
| **110** | -1.57 | -0.1341 | 0.0178 | 0.9965 | 0.0077 | 359650.739 | 0.0073 | 0.7269 | -0.1341 |
| **111** | 0.8676 | 0.0741 | 0.0055 | 1.0091 | 0.0078 | 359708.327 | 0.0073 | 0.7258 | 0.0741 |
| **112** | 0.4573 | 0.038 | 0.0015 | 1.0126 | 0.0078 | 359984.553 | 0.0069 | 0.6914 | 0.038 |
| **113** | 0.3902 | 0.0332 | 0.0011 | 1.0137 | 0.0078 | 359865.051 | 0.0073 | 0.7264 | 0.0332 |
| **114** | 1.1879 | 0.1017 | 0.0103 | 1.0042 | 0.0077 | 359396.478 | 0.0073 | 0.7267 | 0.1017 |
| **115** | -2.3534 | -0.2011 | 0.0391 | 0.9744 | 0.0075 | 232498.029 | 0.0073 | 0.7297 | -0.2011 |
| **116** | 0.1588 | 0.0134 | 0.0002 | 1.0146 | 0.0078 | 359940.252 | 0.0073 | 0.726 | 0.0134 |
| **117** | -1.0269 | -0.088 | 0.0077 | 1.007 | 0.0077 | 359374.789 | 0.0073 | 0.7293 | -0.088 |
| **118** | -0.2804 | -0.0242 | 0.0006 | 1.0143 | 0.0078 | 359991.314 | 0.0073 | 0.7284 | -0.0242 |
| **119** | 0.0452 | 0.0037 | 0 | 1.0149 | 0.0078 | 359895.472 | 0.0073 | 0.7284 | 0.0037 |
| **120** | -0.3682 | -0.0317 | 0.001 | 1.0138 | 0.0078 | 359997.683 | 0.0073 | 0.7276 | -0.0317 |
| **121** | 0.1189 | 0.01 | 0.0001 | 1.0148 | 0.0078 | 359902.047 | 0.0073 | 0.7278 | 0.01 |
| **122** | -1.2413 | -0.105 | 0.011 | 1.0033 | 0.0077 | 359967.948 | 0.0071 | 0.712 | -0.105 |
| **123** | 1.8943 | 0.1608 | 0.0254 | 0.9885 | 0.0076 | 359792.925 | 0.0071 | 0.7116 | 0.1608 |
| **124** | 1.1238 | 0.0958 | 0.0092 | 1.0053 | 0.0077 | 359812.963 | 0.0072 | 0.7208 | 0.0958 |
| **125** | 0.2087 | 0.0176 | 0.0003 | 1.0143 | 0.0078 | 359978.936 | 0.0072 | 0.7161 | 0.0176 |
| **126** | -1.4206 | -0.1216 | 0.0147 | 0.9998 | 0.0077 | 346090.23 | 0.0073 | 0.7297 | -0.1216 |
| **127** | 1.2633 | 0.1082 | 0.0117 | 1.0028 | 0.0077 | 359361.967 | 0.0073 | 0.7266 | 0.1082 |
| **128** | -0.928 | -0.0775 | 0.006 | 1.008 | 0.0078 | 359991.937 | 0.0069 | 0.6924 | -0.0775 |
| **129** | -0.1869 | -0.0162 | 0.0003 | 1.0146 | 0.0078 | 359683.414 | 0.0073 | 0.7296 | -0.0162 |
| **130** | -1.9572 | -0.1673 | 0.0274 | 0.9866 | 0.0076 | 358217.027 | 0.0073 | 0.7288 | -0.1673 |
| **131** | 0.5539 | 0.0459 | 0.0021 | 1.0118 | 0.0078 | 359983.536 | 0.0069 | 0.6856 | 0.0459 |
| **132** | -0.4472 | -0.0383 | 0.0015 | 1.0133 | 0.0078 | 359997.41 | 0.0072 | 0.7229 | -0.0383 |
| **133** | 0.064 | 0.0053 | 0 | 1.0149 | 0.0078 | 359813.401 | 0.0073 | 0.7289 | 0.0053 |
| **134** | 1.9135 | 0.1642 | 0.0264 | 0.9877 | 0.0076 | 358708.121 | 0.0073 | 0.7268 | 0.1642 |
| **135** | -0.187 | -0.0162 | 0.0003 | 1.0146 | 0.0078 | 359940.48 | 0.0073 | 0.7292 | -0.0162 |
| **136** | 0.2911 | 0.0248 | 0.0006 | 1.0142 | 0.0078 | 359805.355 | 0.0073 | 0.728 | 0.0248 |
| **137** | -1.0618 | -0.0908 | 0.0082 | 1.0064 | 0.0077 | 359890.869 | 0.0073 | 0.7267 | -0.0908 |
| **138** | 0.1217 | 0.0102 | 0.0001 | 1.0145 | 0.0078 | 359984.254 | 0.0072 | 0.716 | 0.0102 |
| **139** | 0.3629 | 0.031 | 0.001 | 1.0139 | 0.0078 | 357295.537 | 0.0073 | 0.7296 | 0.031 |

**Supplementary Table 5. Univariate and multi-variable meta-regression.**

| **Variable** | **Results** |
| --- | --- |
| **Geographic region** | **Mixed-Effects Model (k = 139; tau^2 estimator: DL)**  **tau^2 (estimated amount of residual heterogeneity): 0.0009 (SE = 0.0007)**  **tau (square root of estimated tau^2 value): 0.0307**  **I^2 (residual heterogeneity / unaccounted variability): 99.98%**  **H^2 (unaccounted variability / sampling variability): 4972.02**  **R^2 (amount of heterogeneity accounted for): 0.00%**  **Test for Residual Heterogeneity:**  **QE(df = 131) = 651334.2282, p-val < .0001**  **Test of Moderators (coefficients 2:8):**  **F(df1 = 7, df2 = 131) = 4.2888, p-val = 0.0003**  **Model Results:**  **estimate se tval df pval**  **intrcpt 0.0709 0.0402 1.7622 131 0.0804**  **regionEast Asia and Pacific -0.0105 0.0405 -0.2592 131 0.7959**  **regionEurope and Central Asia -0.0183 0.0413 -0.4439 131 0.6579**  **regionLatin America and Caribbean 0.0840 0.0455 1.8459 131 0.0672**  **regionMiddle East and North Africa -0.0160 0.0428 -0.3734 131 0.7094**  **regionNorth America 0.0214 0.0416 0.5147 131 0.6076**  **regionSouth Asia -0.0274 0.0428 -0.6398 131 0.5234**  **regionSub-Saharan Africa -0.0071 0.0444 -0.1593 131 0.8737**  **ci.lb ci.ub**  **intrcpt -0.0087 0.1505 .**  **regionEast Asia and Pacific -0.0906 0.0696**  **regionEurope and Central Asia -0.1001 0.0634**  **regionLatin America and Caribbean -0.0060 0.1741 .**  **regionMiddle East and North Africa -0.1007 0.0688**  **regionNorth America -0.0609 0.1037**  **regionSouth Asia -0.1120 0.0573**  **regionSub-Saharan Africa -0.0948 0.0807**  **---**  **Signif. codes: 0 ‘***’ 0.001 ‘**’ 0.01 ‘*’ 0.05 ‘.’ 0.1 ‘ ’ 1** |
| **Income level** | **Mixed-Effects Model (k = 139; tau^2 estimator: DL)**  **tau^2 (estimated amount of residual heterogeneity): 0.0003 (SE = 0.0003)**  **tau (square root of estimated tau^2 value): 0.0187**  **I^2 (residual heterogeneity / unaccounted variability): 99.95%**  **H^2 (unaccounted variability / sampling variability): 2133.77**  **R^2 (amount of heterogeneity accounted for): 49.83%**  **Test for Residual Heterogeneity:**  **QE(df = 135) = 288058.5115, p-val < .0001**  **Test of Moderators (coefficients 2:4):**  **F(df1 = 3, df2 = 135) = 2.7751, p-val = 0.0438**  **Model Results:**  **estimate se tval df pval ci.lb**  **intrcpt 0.0604 0.0046 13.0188 135 <.0001 0.0512**  **incomeLow income 0.0446 0.0341 1.3065 135 0.1936 -0.0229**  **incomeLower middle income -0.0167 0.0113 -1.4763 135 0.1422 -0.0390**  **incomeUpper middle income 0.0141 0.0081 1.7348 135 0.0851 -0.0020**  **ci.ub**  **intrcpt 0.0696 *****  **incomeLow income 0.1121**  **incomeLower middle income 0.0057**  **incomeUpper middle income 0.0302 .**  **---**  **Signif. codes: 0 ‘***’ 0.001 ‘**’ 0.01 ‘*’ 0.05 ‘.’ 0.1 ‘ ’ 1** |
| **Country development** | **Mixed-Effects Model (k = 139; tau^2 estimator: DL)**  **tau^2 (estimated amount of residual heterogeneity): 0.0003 (SE = 0.0003)**  **tau (square root of estimated tau^2 value): 0.0187**  **I^2 (residual heterogeneity / unaccounted variability): 99.95%**  **H^2 (unaccounted variability / sampling variability): 2108.62**  **R^2 (amount of heterogeneity accounted for): 49.76%**  **Test for Residual Heterogeneity:**  **QE(df = 137) = 288880.7435, p-val < .0001**  **Test of Moderators (coefficient 2):**  **F(df1 = 1, df2 = 137) = 0.6112, p-val = 0.4357**  **Model Results:**  **estimate se tval df pval ci.lb ci.ub**  **intrcpt 0.0605 0.0048 12.6563 137 <.0001 0.0510 0.0699**  **developmentdeveloping 0.0057 0.0073 0.7818 137 0.4357 -0.0088 0.0203**    **intrcpt *****  **developmentdeveloping**  **---**  **Signif. codes: 0 ‘***’ 0.001 ‘**’ 0.01 ‘*’ 0.05 ‘.’ 0.1 ‘ ’ 1** |
| **Publication year** | **Mixed-Effects Model (k = 139; tau^2 estimator: DL)**  **tau^2 (estimated amount of residual heterogeneity): 0.0007 (SE = 0.0005)**  **tau (square root of estimated tau^2 value): 0.0263**  **I^2 (residual heterogeneity / unaccounted variability): 99.98%**  **H^2 (unaccounted variability / sampling variability): 5325.10**  **R^2 (amount of heterogeneity accounted for): 0.86%**  **Test for Residual Heterogeneity:**  **QE(df = 137) = 729538.6736, p-val < .0001**  **Test of Moderators (coefficient 2):**  **F(df1 = 1, df2 = 137) = 1.6334, p-val = 0.2034**  **Model Results:**  **estimate se tval df pval ci.lb ci.ub**  **intrcpt 0.0607 0.0045 13.5687 137 <.0001 0.0518 0.0695 *****  **publicationbefore 0.0102 0.0080 1.2780 137 0.2034 -0.0056 0.0259**  **---**  **Signif. codes: 0 ‘***’ 0.001 ‘**’ 0.01 ‘*’ 0.05 ‘.’ 0.1 ‘ ’ 1** |
| **Study size** | **Mixed-Effects Model (k = 139; tau^2 estimator: DL)**  **tau^2 (estimated amount of residual heterogeneity): 0.0007 (SE = 0.0005)**  **tau (square root of estimated tau^2 value): 0.0263**  **I^2 (residual heterogeneity / unaccounted variability): 99.98%**  **H^2 (unaccounted variability / sampling variability): 5363.58**  **R^2 (amount of heterogeneity accounted for): 0.73%**  **Test for Residual Heterogeneity:**  **QE(df = 137) = 734810.2003, p-val < .0001**  **Test of Moderators (coefficient 2):**  **F(df1 = 1, df2 = 137) = 12.9431, p-val = 0.0004**  **Model Results:**  **estimate se tval df pval ci.lb ci.ub**  **intrcpt 0.0756 0.0048 15.6996 137 <.0001 0.0660 0.0851 *****  **size>10000 -0.0257 0.0071 -3.5977 137 0.0004 -0.0398 -0.0116 *****  **---**  **Signif. codes: 0 ‘***’ 0.001 ‘**’ 0.01 ‘*’ 0.05 ‘.’ 0.1 ‘ ’ 1** |
| **Study quality** | **Mixed-Effects Model (k = 139; tau^2 estimator: DL)**  **tau^2 (estimated amount of residual heterogeneity): 0.0010 (SE = 0.0007)**  **tau (square root of estimated tau^2 value): 0.0316**  **I^2 (residual heterogeneity / unaccounted variability): 99.98%**  **H^2 (unaccounted variability / sampling variability): 5175.60**  **R^2 (amount of heterogeneity accounted for): 0.00%**  **Test for Residual Heterogeneity:**  **QE(df = 137) = 709056.7855, p-val < .0001**  **Test of Moderators (coefficient 2):**  **F(df1 = 1, df2 = 137) = 0.0709, p-val = 0.7904**  **Model Results:**  **estimate se tval df pval ci.lb ci.ub**  **intrcpt 0.0638 0.0042 15.3793 137 <.0001 0.0556 0.0720 *****  **qualitysmall 0.0026 0.0097 0.2663 137 0.7904 -0.0167 0.0219**  **---**  **Signif. codes: 0 ‘***’ 0.001 ‘**’ 0.01 ‘*’ 0.05 ‘.’ 0.1 ‘ ’ 1** |
| **Multi-variable meta regression** | **Multimodel Inference: Final Results**  **--------------------------**  **- Number of fitted models: 64**  **- Full formula: ~ region + development + size + quality + income + publication**  **- Coefficient significance test: knha**  **- Interactions modeled: no**  **- Evaluation criterion: AICc**  **Best 5 Models**  **--------------------------**  **Global model call: metafor::rma(yi = TE, sei = seTE, mods = form, data = glm.data,**  **method = method, test = test)**  **---**  **Model selection table**  **(Intrc) dvlpm incom pblct qulty size df logLik AICc delta weight**  **33 + + 3 237.571 -469.0 0.00 0.582**  **35 + + + 6 240.286 -467.9 1.03 0.348**  **37 + + + 4 235.434 -462.6 6.40 0.024**  **34 + + + 4 235.414 -462.5 6.44 0.023**  **41 + + + 4 235.388 -462.5 6.49 0.023**  **Models ranked by AICc(x)**  **Multimodel Inference Coefficients**  **--------------------------**  **Estimate Std. Error z value Pr(>\|z\|)**  **intrcpt 7.723500e-02 0.0058750942 13.146173069 0.0000000**  **size>10000 -2.922071e-02 0.0093599144 3.121899255 0.0017969**  **incomeLow income 1.633925e-02 0.0284679902 0.573951688 0.5660005**  **incomeLower middle income -1.247740e-02 0.0181444870 0.687669016 0.4916612**  **incomeUpper middle income 6.326754e-03 0.0103121712 0.613522957 0.5395306**  **publicationbefore 2.840661e-04 0.0022200824 0.127952951 0.8981862**  **developmentdeveloping -1.117681e-04 0.0041589808 0.026873918 0.9785603**  **qualitysmall 7.165686e-05 0.0018323350 0.039106853 0.9688052**  **regionEast Asia and Pacific -5.156076e-06 0.0006700936 0.007694561 0.9938607**  **regionEurope and Central Asia -6.701545e-06 0.0007395521 0.009061626 0.9927700**  **regionLatin America and Caribbean 1.109478e-05 0.0010041800 0.011048600 0.9911847**  **regionMiddle East and North Africa -9.788119e-06 0.0009154044 0.010692672 0.9914686**  **regionNorth America 1.316354e-06 0.0005893351 0.002233626 0.9982178**  **regionSouth Asia -1.235636e-05 0.0010525393 0.011739568 0.9906334**  **regionSub-Saharan Africa -7.702168e-06 0.0008291142 0.009289635 0.9925881**  **Predictor Importance**  **--------------------------**  **model importance**  **1 size 0.9778970372**  **2 income 0.3682720536**  **3 publication 0.0465027805**  **4 development 0.0385430457**  **5 quality 0.0354393885**  **6 region 0.0002106707** |

**Supplementary Figure 1. Sensitivity analysis by using a build-in function.**

**
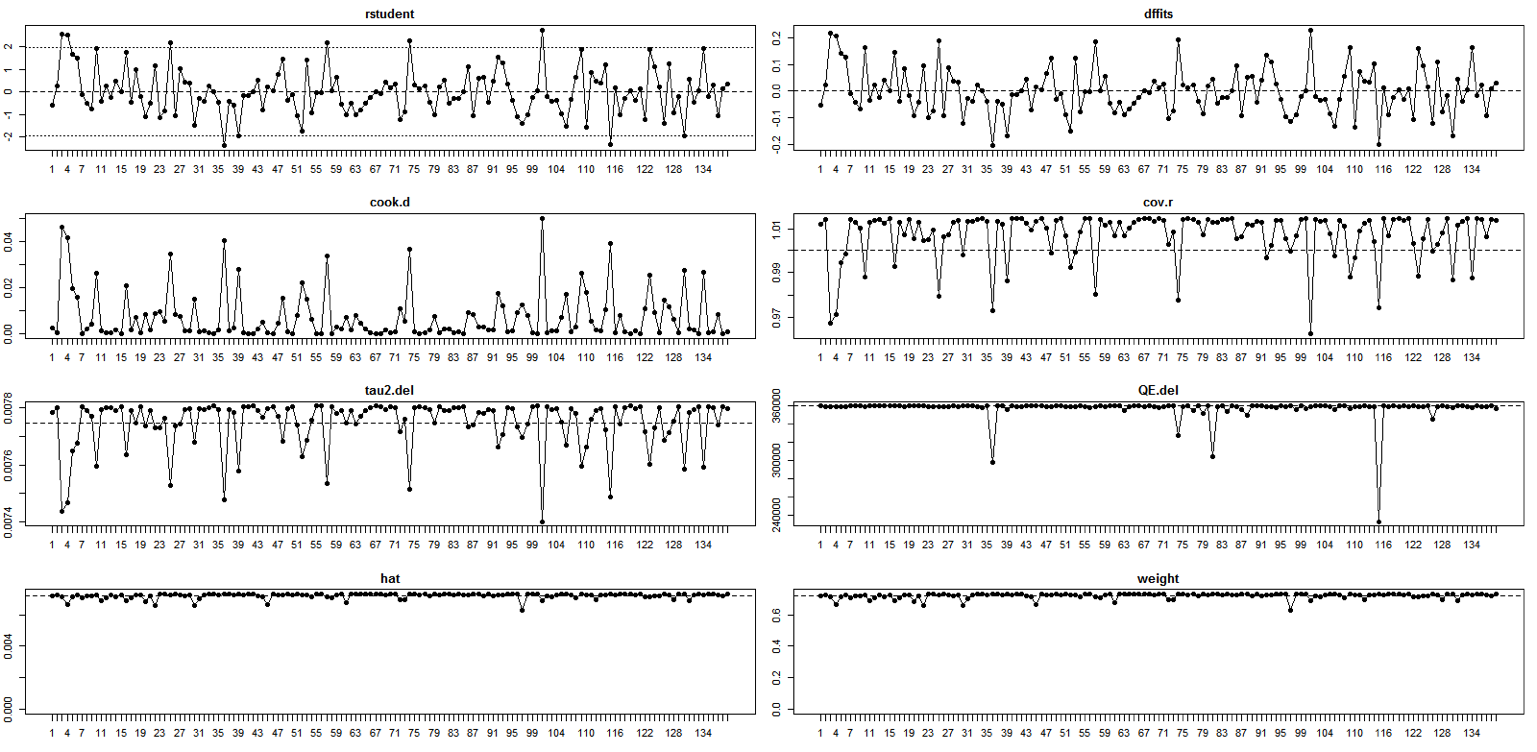
**
